# Lineage-specific evolution and resistance-virulence divergence in *Klebsiella pneumoniae* ST268: a global population genomic analysis

**DOI:** 10.1128/aac.00703-25

**Published:** 2025-09-15

**Authors:** Xu Dong, Yanghui Xiang, Yi Li, Ying Zhang

**Affiliations:** 1 State Key Laboratory for Diagnosis and Treatment of Infectious Diseases, National Clinical Research Center for Infectious Diseases, China-Singapore Belt and Road Joint Laboratory on Infection Research and Drug Development, National Medical Center for Infectious Diseases, Collaborative Innovation Center for Diagnosis and Treatment of Infectious Diseases, The First Affiliated Hospital, Zhejiang University School of Medicine 71069, Hangzhou, China; 2 Yuhang Institute for Collaborative Innovation and Translational Research in Life Sciences and Technology 661980 , Hangzhou, China; Johns Hopkins University School of Medicine, Baltimore, Maryland, USA

**Keywords:** *Klebsiella pneumoniae*, ST268, hypervirulence, carbapenem-resistance, emerging pathogen

## Abstract

*Klebsiella pneumoniae* sequence type 268 (ST268) represents an emerging high-risk clone with significant clinical implications. Here, we present a comprehensive genomic analysis of 562 ST268 isolates collected from 44 countries across six continents between 2001 and 2024. Phylogenomic reconstruction revealed 10 distinct lineages with diverse geographic and functional signatures. Bayesian analysis dated the emergence of ST268 to ~1,908 (95% confidence interval [CI]: 1,841–1,952), with Europe as the likely origin. Our findings uncovered a clear evolutionary split between hypervirulent and multidrug-resistant subclones. Lineages L1 and L3 were enriched for carbapenemase genes (66.1% and 60%) and acquired genes involved in inorganic ion transport and amino acid metabolism, while L2 and L6 showed preferential carriage of hypervirulence markers and gene acquisitions related to secretion systems. Plasmid profiling revealed distinct virulence and resistance plasmid populations, with notable co-occurrence in 48 isolates predominantly from lineages L2 and L6, yet minimal integration between these elements. Nonsynonymous single-nucleotide polymorphisms further revealed lineage-specific functional variations, potentially driven by distinct ecological pressures. Phenotypic validation in *Galleria mellonella* and mouse models confirmed the high virulence of L2 and L6 strains. These patterns suggest a functional trade-off between resistance and virulence among ST268 lineages, likely shaped by parallel evolution under selective constraints. Our study provides novel insights into the genomic architecture and adaptive divergence of ST268, highlighting the need for lineage-informed surveillance and intervention strategies to mitigate its ongoing global dissemination.

## INTRODUCTION

*Klebsiella pneumoniae*, a prominent member of the ESKAPE pathogens (1), is a leading cause of hospital- and community-acquired infections, including respiratory, urinary tract, and bloodstream infections (2). Traditionally, *K. pneumoniae* has been classified into two major pathotypes: classical *K. pneumoniae* (cKP) and hypervirulent *K. pneumoniae* (hvKP) strains (3, 4). While cKP strains are typically multidrug-resistant (MDR) and associated with nosocomial infections, hvKP strains are generally antibiotic-sensitive but capable of causing invasive and often life-threatening infections in healthy individuals (3, 5). Although the string test was historically employed to identify hvKP, its limited specificity (6, 7) has prompted a shift toward molecular detection. Recent research has established that hypervirulence (hv) in *K. pneumoniae* is primarily driven by key functional genes, particularly those encoding the aerobactin (*iucA*) (8) and salmochelin (*iroB*) siderophore systems (9), as well as regulators of the mucoid phenotype (*rmpA*/*rmpA2*) (10). In addition, *peg-344*—which encodes a putative metabolite transporter—although not universally required for full virulence (11), is frequently co-located with these loci and thus serves as a useful genomic marker for hypervirulent strains (12, 13).

Despite the increasing research on cKP and hvKP lineages, such as ST11 and ST23 (14–16), certain genetic lineages, such as sequence type 268 (ST268), remain strikingly under-characterized. ST268 has been classified as part of clonal group 36 (CG36), a genetically distinct group positioned at the interface between hypervirulent and multidrug-resistant clones (17). Originally detected in a retrospective Norwegian study from 2001 (18), ST268 has since been sporadically reported in both clinical and environmental contexts. For instance, non-epidemic ST268 isolates harboring *rmpA2* were identified in a longitudinal study in Malawi (19), while a recent investigation in northern Japan surprisingly found ST268 to be the dominant sequence type (ST) among local hvKP isolates, frequently carrying CTX-M-type extended-spectrum β-lactamase (ESBL) genes (20). Similarly, reports from China have documented ST268 strains simultaneously harboring carbapenem resistance and hypervirulence determinants (21, 22), highlighting the concerning convergence of these traits. Moreover, ST268 has been recovered from water sources in Germany (23), and carbapenem-resistant variants have been isolated from sewage (24), suggesting a potentially overlooked role in environmental transmission and antimicrobial resistance (AMR) dissemination.

Despite scattered reports, the evolutionary dynamics, genomic diversity, and global epidemiology of ST268 remain poorly resolved. Given its presence in both hypervirulent and drug-resistant contexts, ST268 may represent an emerging convergent lineage blending traits of both pathotypes, posing a significant public health threat. To address this gap, we performed the first comprehensive genomic analysis of this lineage, systematically examining genomes of global ST268 *K. pneumoniae* isolates to delineate their population structure, genetic diversity, and evolutionary history. Through robust comparative genomic and phylogenetic approaches, we revealed that ST268 has diverged into distinct hypervirulent and multidrug-resistant subclones and identified lineage-specific genomic markers underlying its ecological adaptability and clinical relevance. Together, our findings elucidate the complex biology of this understudied yet potentially high-risk clone, underscoring the urgent need for enhanced genomic surveillance and global monitoring of *K. pneumoniae* ST268.

## MATERIALS AND METHODS

### Public data set collection, reassembly, and quality control

All *K. pneumoniae* genomes were retrieved from the NCBI GenBank database as of 31 December 2024. Next, we searched the AllTheBacteria database (25), which contains 661 k Sequence Read Archive (SRA) data sets, using the keyword “*K. pneumoniae*” to extract relevant metadata, and downloaded all corresponding pre-assembled SRA genomes. Kleborate v3.1.2 (26) was employed to determine the species classification and multi-locus sequence typing (MLST) of each genome, with a particular focus on isolates identified as ST268. One-locus variants (ST268_1LV) were also included, as they are indistinguishable from ST268 in phylogenetic analyses (Fig. S1); hence, they are collectively referred to as ST268 hereafter.

For the SRA assemblies classified as ST268, the original SRA data were additionally downloaded using SRA Toolkit (https://github.com/ncbi/sra-tools) and reassembled using the fq2dna pipeline (https://gitlab.pasteur.fr/GIPhy/fq2dna). The quality of all assemblies was evaluated using Checkm v1.2.2 (27) and Checkm2 v1.0.2 (28). For genomes available from both GenBank and SRA, the assembly with the higher N50 value was selected. Genomes that did not meet our quality thresholds (contigs <300, N50 >50 kb, completeness >90%, and contamination <5%) were omitted from further analyses, yielding 558 unique public genomes.

### Strains and whole-genome sequencing

Between January 2022 and December 2024, a total of four *K. pneumoniae* isolates identified as ST268 were recovered from patients at the First Affiliated Hospital of Zhejiang University School of Medicine (Hangzhou, China). These were clinical isolates obtained from blood (kpn567), bronchoalveolar lavage fluid (kpn976), and sputum (kpn44 and kpn402) samples of patients diagnosed with bloodstream infection or pneumonia in the intensive care unit or emergency department. Genomic DNA was extracted using the QIAamp DNA Mini Kit (Qiagen, Hilden, Germany). Sequencing libraries were prepared using the Rapid Plus DNA Library Prep Kit for Illumina V2 (ABclonal, RK20255, Wuhan, China) according to the manufacturer’s instructions. Whole-genome sequencing was conducted on an Illumina NovaSeq 6000 platform to generate paired-end reads (2 × 150 bp). Genome assemblies were produced using the fq2dna pipeline. Additionally, two strains were selected for long-read sequencing on the Oxford Nanopore MinION platform. Hybrid assemblies of the complete genomes were achieved by integrating Illumina short-read and Nanopore long-read data using either Unicycler v0.5.1 (29) or Canu v2.2 (30). Annotation of assemblies was used bakta v1.8.2 (31). Pan-genome analysis was performed using Panaroo v1.3.2 (32) with the GFF3 file generated by batka as the input file. Additionally, genes were annotated with Clusters of Orthologous Groups (COG) functional categories using eggNOG-mapper v2.1.9 (33). The Tajima’s *D* statistic was calculated for each core gene of the ST268 using the PopGenome package (34). Virulence and antimicrobial resistance scores, as well as yersiniabactin (*ybt*) lineage and ICE*Kp* structure typing, were determined using Kleborate, while capsule (K) and lipopolysaccharide (LPS; O) locus types were identified using Kaptive v3.0.0b6 (35).

### Population structure and phylogenetic construction

The complete genome of the KP161637 (accession no: CP123043) (36) was used as the reference genome for ST268. Reference-based mapping and single-nucleotide polymorphisms (SNPs) identification within the ST268 core genome were performed using Snippy v4.6.0 (https://github.com/tseemann/snippy). Recombined regions in the core genome were detected and excluded using Gubbins v3.2.1 (37). This recombination-free SNP output was then used as input to reconstruct the phylogeny using IQtree (v2.3.6) (38) with 10,000 ultrafast bootstrap replicates, and the best fitted model (GTR + F + ASC + R2) was selected by ModelFinder (39). The geographic origins were deduced using the Bayesian binary Markov chain Monte Carlo (MCMC) method in RASP v4.2 (40), employing 10 parallel chains each running for 50 million cycles. Lineages within the maximum likelihood (ML) tree were delineated using FastBaps v1.0.8 (41), with the best_baps_partition function incorporating the phylogeny as a prior. A minimum spanning tree was constructed to visualize the genetic relationships among all ST268 isolates using PHYLOViZ v2.0 (42).

### Bayesian phylogenetic inference

For constructing a timed phylogeny, a subset of 144 genomes was selected to represent the phylogenetic diversity as well as the full range of isolation dates and geographic origins. The temporal signal within this data set was initially evaluated using a root-to-tip regression analysis in TempEst v1.5.3 (43). To further validate the temporal signal, the TipDatingBeast package (44) was employed, with the sampling dates randomly shuffled 20 times under the same parameters as the original data set.

BEAST v1.8.4 (45) was used to estimate both the substitution rate and the timing of the most recent common ancestor (MRCA) under a GTR substitution model, an optimized relaxed clock model with an uncorrelated lognormal distribution, and the Bayesian Skyline population model, as determined by path sampling (46). Four independent MCMC chains were run for 2 × 10^8^ generations each, with samples drawn every 1,000 generations. A 10% burn-in was removed from each chain before combining the runs to generate the consensus tree. Convergence was confirmed using Tracer v1.7.2 (47), ensuring that all essential parameters achieved effective sample sizes greater than 200. The estimation of effective population size (EPS) dynamics was performed with the skygrowth package in R (48).

### Identification of antimicrobial resistance genes, virulence factors, and prophages

Genetic determinants of AMR were identified using AMRFinderPlus v4.0.19 (49) with minimum coverage of 90% and identity threshold of 90%, while virulence factors were detected using ABRicate v1.0.0 (https://github.com/tseemann/abricate) against the Virulence Factor Database (VFDB)(50). The *peg-344* gene (NC_005249) was detected via BLASTn searches of the draft genome assemblies using a minimum identity threshold of 70%. For genes of interest exhibiting low coverage, SRST2 v0.2.0 (51) was additionally employed against reference sequences to confirm their integrity. Hv-CRKP isolates were defined as those positive for at least one of these hypervirulence genes (*rmpA*, *rmpA2*, *peg-344*, *iucA*, and *iroB*) (14). Prophage regions were predicted using the online tool PHASTEST (52).

### Plasmid analysis

As described previously (53), plasmid contigs were identified and reconstructed using the mob-recon tool from the MOB-suite toolkit v3.1.2, used with default settings (54). To assess sequence relationships among plasmids, we performed clustering using Mge-cluster v1.1.0 (55) with the parameters “--perplexity 100 min_cluster 10.” This tool groups plasmids based on unitig (56) presence/absence profiles, with dimensionality reduction and visualization performed via the t-distributed stochastic neighbor embedding (t-SNE) algorithm implemented in the openTSNE library (57), as integrated in Mge-cluster.

### Detection of clade-specific SNPs

The SNPs associated with isolates of each cluster were detected using Scoary v1.6.16 (58).

### Virulence assays

Animal experiments were performed in accordance with the approval granted by the Institutional Animal Care and Ethics Committee of the First Affiliated Hospital of Zhejiang University, School of Medicine (reference number: 2023-938). We employed both *Galleria mellonella* and mouse intraperitoneal infection models as described in previous studies (59). Briefly, 10 larvae, each approximately 250 mg, were randomly chosen and tested for each isolate. A 20 µL bacterial suspension (10^6^ CFU/mL in phosphate-buffered saline [PBS]) was injected into the second left proleg, followed by incubation at 37°C and observation every 24 hours. In the mouse model, 5-week-old female BALB/c mice (10 per group) received an intraperitoneal injection of 1 × 10^7^ CFU of each isolate in 100 µL PBS. Post-inoculation, the mice’s physical condition was assessed and documented at 24-hour intervals. As controls, the hypervirulent *K. pneumoniae* strain NTUH-K2044 and the environmentally derived strain *K. pneumoniae* CGMCC1.839 were used as control of high and low virulence, respectively.

### Statistical analysis

Unless otherwise specified, all bioinformatic tools were executed using their default parameters. Statistical comparisons were carried out employing either the *χ*^2^ test or Fisher’s exact test, supplemented by the Wilcoxon rank-sum test where appropriate. A *P*-value of less than 0.05 was considered indicative of statistical significance. Data processing and visualization were performed using R (version 4.2.1; https://www.r-project.org/).

## RESULTS

### General characteristics of ST268

**Fig 1:**
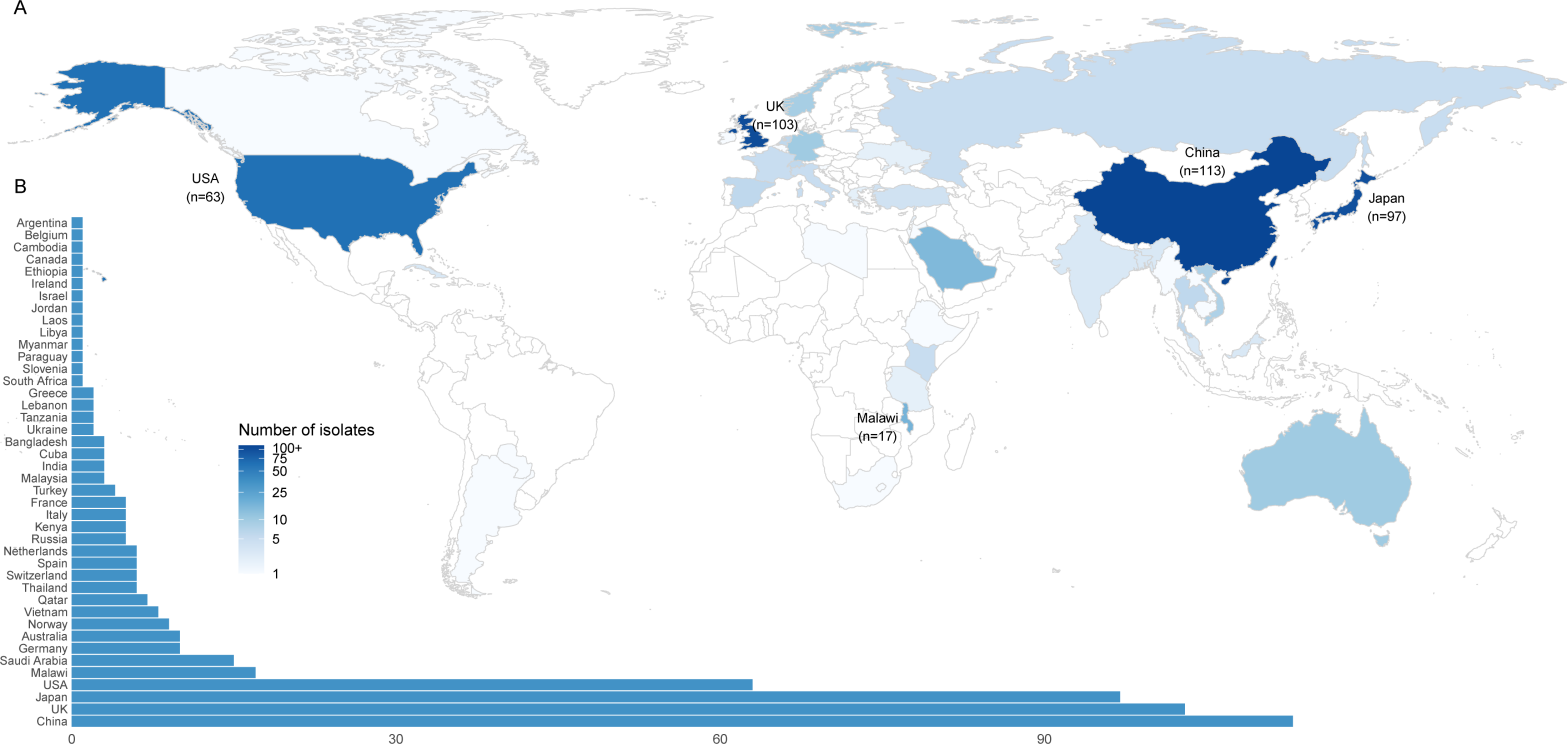
Geographical distribution of ST268 isolates. (**A**) Global map illustrating the geographic distribution of ST268 isolates. (**B**) Bar chart displaying the number of ST268 strains identified across various countries.

A total of 562 ST268 strains were collected from 44 countries across six continents between 2001 and 2024, with the majority isolated from China (*n* = 113), followed by the United Kingdom (*n* = 103), Japan (*n* = 97), and the United States (*n* = 63; Fig. 1). Human clinical samples constituted the primary source (85.2%, 479/562), with additional isolates recovered from hospital environments (7.8%, 44/562), environmental sources (1.1%, 6/562), animals (0.5%, 3/562), and food (0.18%, 1/562). The sources of 29 isolates (5.2%) were unidentified. Detailed isolate information is provided in Table S1.

### Population structure of ST268

**Fig 2:**
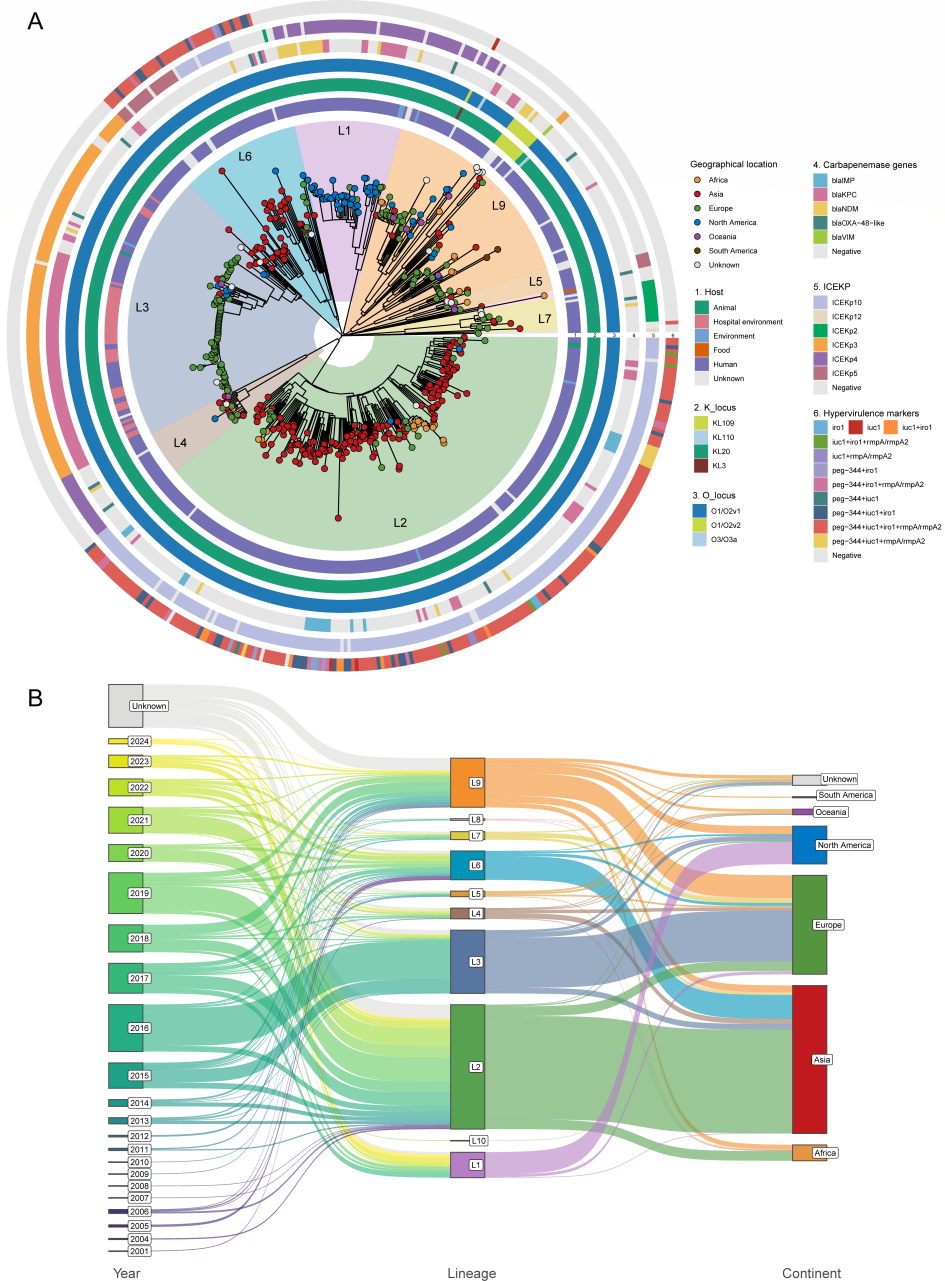
Core-genome SNP phylogeny and spatiotemporal dynamics of ST268 population. (**A**) ML phylogenetic tree of 562 ST268 isolates, rooted using an outgroup (not shown), and constructed from recombination-filtered core-genome SNPs. Isolate characteristics are indicated by the color legend. Due to their minimal representation, lineages L8 and L10 are not individually labeled. (**B**) A mulberry diagram illustrating the relationship between strain isolation dates, lineages, and continental origins.

An ML phylogenetic tree was constructed based on 13,961 single-nucleotide variants identified within the non-repetitive, non-recombinant core genome of 562 isolates (Fig. 2A; Fig. S2). Six clusters could be clearly identified based on the topology of the phylogenetic tree (Fig. S3). To further resolve the population structure, we applied FastBaps clustering and selected level 1 among the hierarchical outputs, resulting in the delineation of 10 lineages (L1–L10; Fig. S4). Lineage L2 emerged as the predominant group, comprising 39.1% (220/562) of all isolates. Geographic specificity was observed among several lineages, with L2 and L6 predominantly found in Asia, L3 in Europe, and L1 primarily in North America (Fig. 2B). The median SNP distance between lineages was 249 (range: 166–428), while the overall pairwise SNP distances among ST268 isolates ranged from 0 to 428. Using a 16-SNP threshold to define transmission clusters (60), we identified 75 independent transmission events, including three major events involving more than 10 strains—two from lineage L3 representing clonal outbreaks in separate UK hospitals (61, 62) and one from lineage L9 spanning three continents (Fig. S5).

All lineages exhibited consistent capsule synthesis loci (KL20) and LPS biosynthesis loci (O1/O2v1), except for lineage L9, which displayed greater diversity with additional K loci (KL109, KL110, and KL3) and O loci (O1/O2v2 and O3/O3a). The integrative conjugative element (ICE*Kp*) encoding *ybt* and its receptor was detected in 80.4% (452/562) of isolates, with distinct lineage-specific distribution patterns: ICE*Kp10* (*ybt0*) predominated in L2 (95.5%, 210/220), ICE*Kp4* (*ybt10*) in both L1 (91.1%, 41/45) and L4 (100%, 19/19), and ICE*Kp3* (*ybt9*) in L3 (96.4%, 108/112). Lineage L7 predominantly harbored ICE*Kp2* (*ybt10*; 85.7%, 12/14), while lineages L6 and L9 showed greater diversity in ICE*Kp* types, with L6 primarily associated with ICE*Kp5* (*ybt14*, 35.3%) and ICE*Kp10* (*ybt0*, 31.4%) and L9 most commonly carrying ICE*Kp4* (*ybt10*, 23.0%; Fig. 2A; Table S2).

### Recombination events among ST268 isolates

**Fig 3:**
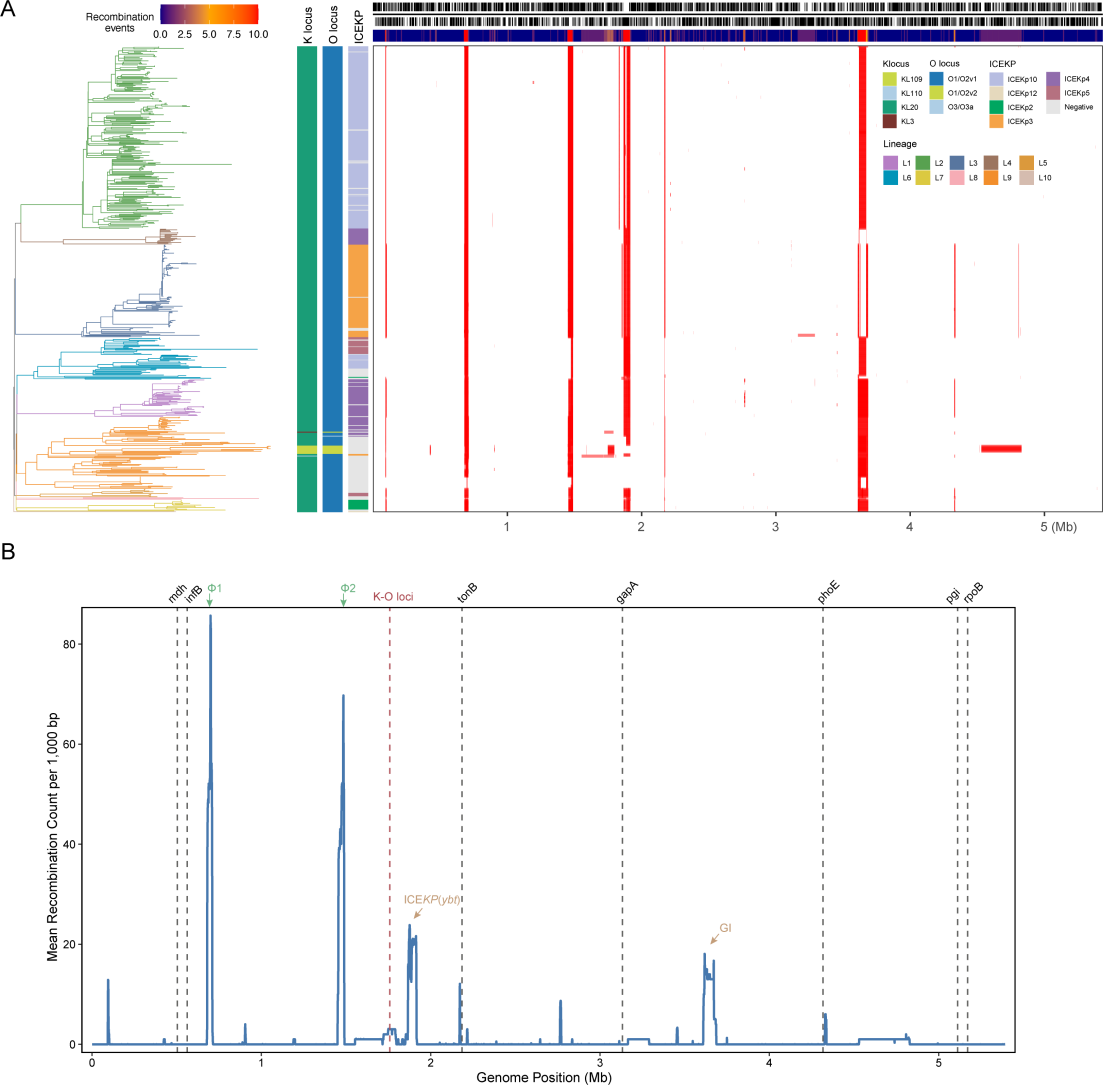
Genome-wide recombination analysis across ST268 lineages. (**A**) The left panel displays the core-genome SNP-based phylogenetic tree from Fig. 2, with branches colored according to the 10 identified lineages. The corresponding right panel illustrates the genomic locations of detected recombination events (red blocks) across all isolates, identified using Gubbins. (**B**) Recombination frequency histogram showing the distribution of recombination events across the ST268 chromosome, calculated over non-overlapping 1,000 bp windows. Gray vertical lines mark the positions of seven MLST loci used for sequence typing. Peaks in the distribution indicate potential recombination hotspots.

Recombination, a major driver of bacterial evolution, plays a critical role in the genetic diversification of *K. pneumoniae* ST types (63–65). To investigate the role of recombination in the evolution of ST268, we analyzed the entire genome data set using Gubbins. We identified 617 putative recombination events (Fig. 3A and B). Of these, three (0.4%) spanned large regions (>100 kbp), including a 161-kbp event encompassing the K and O loci, where the integrative and conjugative element (ICE) element encoding *ybt* locus marked a key recombination hotspot (Fig. 3B). Two additional phage-related hotspots were detected: Φ1 as *Klebsiella* phage ST15_OXA48phi14.1 (NC_049454) and Φ2 as *Salmonella* phage 118970_sal3 (NC_031940; Fig. 3B). Additionally, another recombination hotspot was observed around ~3.6 Mbp (Fig. 3B), corresponding to a putative genomic island (GI) enriched in genes encoding mobile genetic elements, including multiple transposases and recombinases, and containing a tetrathionate respiration (*ttr*) gene cluster.

### Time scale of the emergence of global ST268 isolates

**Fig 4:**
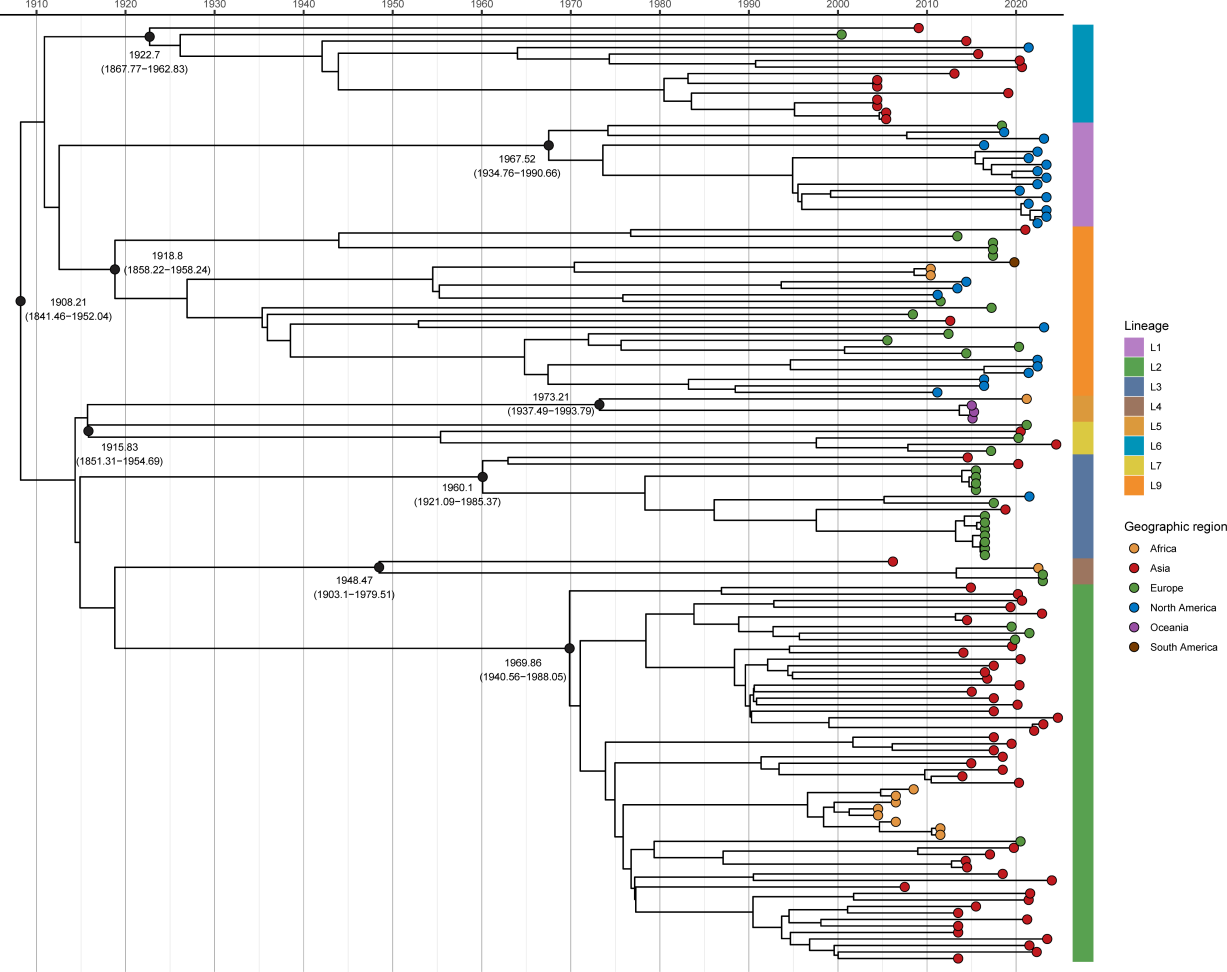
A maximum clade credibility tree, generated using BEAST, displays ST268 isolates with tips colored by geographic origin (continent).

To estimate the origin of ST268, we first confirmed a strong temporal signal in our data set (Fig. S6). A Bayesian phylogenetic analysis of 144 spatially and temporally representative isolates estimated a substitution rate of 4.13 × 10^−7^ substitutions per site per year (95% highest posterior density: 2.28 × 10^−7^ to 6.23 × 10^−7^), resulting in an MRCA for all ST268 isolates dating to approximately 1,908 (95% CI, 1,841–1,952). Distinct lineages within ST268 emerged progressively throughout the early to mid-20th century (Fig. 4).

A median Tajima’s *D* of −1.36 across core genes suggests recent population expansion or a potential selective sweep within ST268. The EPS of ST268, inferred using a Bayesian skyline model, showed a potential trend of slow initial growth, followed by a possible expansion after the year 2000 and a subsequent stabilization. However, the wide 95% credible intervals indicate considerable uncertainty in these estimates (Fig. S7). Furthermore, Bayesian phylogeographic analysis indicated Europe as the most probable geographical origin of the ST268 lineage, with subsequent global dissemination from this ancestral population (Fig. S8).

### Prevalence and distribution of carbapenemases, ESBLs, and hypervirulence markers

Acquired resistance genes, notably carbapenemases and ESBLs, alongside hv markers, complicate *K. pneumoniae* control. We examined the distribution of these determinants across ST268 lineages using Kleborate, which revealed an average virulence score of 2.6 and a resistance score of 0.9, with notable variation among lineages. Specifically, lineage L2 exhibited the highest virulence score (mean: 4.6), followed by L6 (3.9), while lineages L3 and L1 showed the highest resistance scores (1.5 and 1.3, respectively; Fig. S9).

**Fig 5:**
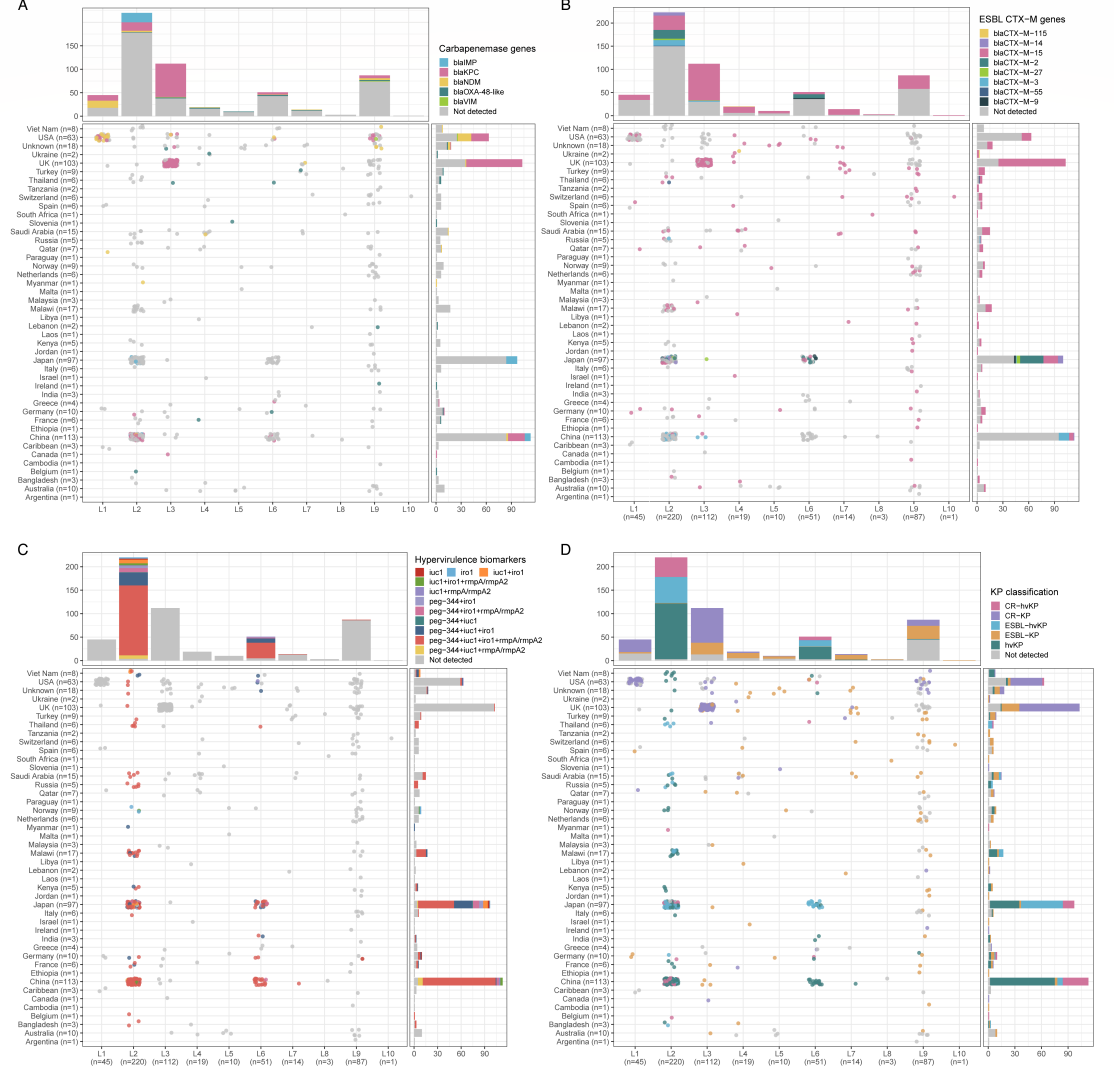
Distribution of major antibiotic resistance and hv markers by ST268 lineages and countries. Scatter plots summarize the distribution of (**A**) carbapenemase genes, (**B**) ESBL CTX-M genes, (**C**) hv marker genes, and (**D**) strain classification. Each circle represents a genome, colored according to the presence or combination of marker genes. Bar plots above and on the right display the percentage of genomes per lineage and country, respectively, with colors corresponding to marker gene combinations.

Carbapenemase genes were detected in 30.2% (170/562) of isolates, with *bla*_KPC_ (predominantly *bla*_KPC-2_) being most prevalent (65.9%, 112/170), followed by *bla*_IMP_ (11.8%, 20/170), *bla*_NDM_ (10.4%, 26/170), *bla*_OXA-48_-like (6.5%, 11/170), and *bla*_VIM_ (0.6%, 1/170). All lineages except L8 and L10 harbored carbapenemase genes, with detection rates ranging from 14.3% to 66.1%. Lineages L1 and L3 showed the highest prevalence (66.1% and 60%, respectively). Geographically, isolates from the United Kingdom and the United States demonstrated particularly high carbapenemase carriage (66.0% and 60.3%, respectively; Fig. 5A).

ESBL genes, primarily CTX-M variants, were more common, present in 243 isolates (43.2%), with CTX-M-15 dominating (187, 77.0%). Detection rates across lineages ranged from 24.4% to 100%, with L7 leading among major lineages (≥10 isolates) at 85.7%, followed by L3 (73.2%) and L4 (70%). Unexpectedly, L1 showed the lowest rate (24.4%, 11/45; Fig. 5B).

Hv markers—*iutA*, *iroB*, *rmpA/rmpA2*, and *peg-344*—were identified in 47.0% (264/562) of isolates. Co-occurrence of all four markers was the predominant pattern (69.3%, 183/264). The distribution of hv markers was highly lineage specific, concentrated primarily in L2 and L6, with only isolated occurrences in L7 and L9. Geographically, isolates from Japan (95.9%), China (95.6%), and Malawi (82.4%) showed the highest prevalence of hv markers (Fig. 5C).

### Comprehensive genomic analysis of antimicrobial resistance and virulence determinants

**Fig 6:**
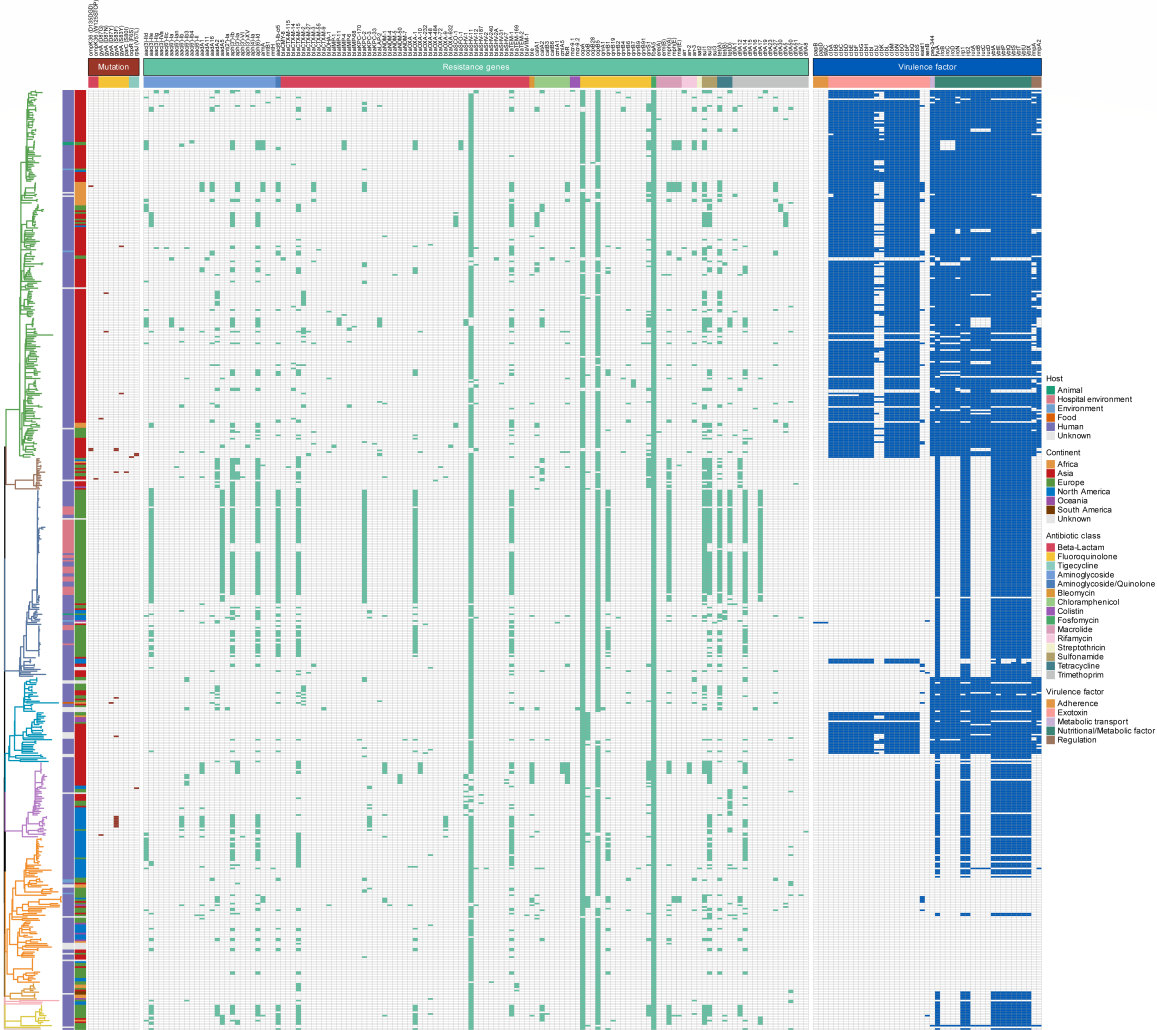
A heatmap illustrates the distribution of mutations, ARGs, and non-core virulence genes across ST268 lineages. Squares colored by trait category indicate the presence of each examined trait.

Beyond the resistance and hypervirulence determinants described above, we analyzed the AMR and virulence gene profiles of all 562 ST268 isolates to provide a comprehensive overview. Genomic analysis identified a highly conserved set of chromosomal resistance genes (Fig. 6), including the quinolone efflux pumps *oqxA* (97.2%, 546/562) and *oqxB12* (91.5%, 514/562), the fosfomycin resistance gene *fosA5* (100%), and the β-lactamase gene *bla*_SHV-11_ (92.3%, 519/562). When integrated with the phylogenetic tree, these results revealed a consistent distribution pattern of resistance genes in lineage L3, aligning with the transmission events previously observed, including clonal outbreaks in UK hospitals. Moreover, lineage L3 exhibited the highest number of acquired antibiotic resistance genes (ARGs; median: 16.5, *P* < 0.05, except for L7; Fig. S10A), and isolates from Europe carried more acquired ARGs than those from other continents (median: 10, *P* < 0.01, except for Africa; Fig. S10B). Notably, mutations associated with AMR were infrequent across all ST268 lineages (Fig. 6).

We then evaluated the complete set of virulence genes to assess the pathogenic potential of the isolates. To focus on genes indicative of variable pathogenicity, we excluded highly conserved virulence genes present in >95% of strains. To ensure statistical independence, genes within known operons (e.g., yersiniabactin and colibactin) were counted once as independent virulence factors. Consistent with the hv-marker analysis, lineage L2 carried the highest number of variable virulence loci (median = 6, *P* < 0.01; Fig. S10C). In contrast, within lineage L6, only the ICE*Kp10*-positive clade possessed the colibactin gene cluster (Fig. 6). Additionally, isolates from Asia and Africa harbored more variable virulence loci (medians = 6 and 6, respectively, *P* < 0.01) than those from other continents (Fig. S10D).

### Plasmid characterization and dynamics

To better understand the dynamics of plasmids in the ST268 isolates, we reconstructed plasmids from strains carrying carbapenemase genes or hv markers using MOB-suite (Table S3). Of 170 carbapenemase-positive isolates, 164 (96.5%) yielded reconstructed plasmids linked to 20 replicon types, which refer to incompatibility (Inc) groups classified based on plasmid replication and partitioning systems. The *bla*_KPC_-carrying plasmids exhibited the greatest diversity (13 replicon types), followed by *bla*_NDM_-carrying plasmids (eight types). The *bla*_KPC_ genes were predominantly located on multi-replicon IncFIB/IncHI1B plasmids (53.2%, 59/111), while *bla*_NDM_ genes were primarily found on IncC plasmids (40.9%, 9/22). The *bla*_IMP_- and *bla*_OXA-48_-like genes were most frequently associated with IncN (40%, 8/20) and IncL/M (60%, 6/10) plasmids, respectively (Fig. S11). Among the 269 isolates harboring hypervirulence markers, we reconstructed 262 virulence plasmids, predominantly of IncFIB type (*n* = 203), followed by IncFIB/IncHI1B multi-replicon plasmids (*n* = 46; Fig. S12).

**Fig 7:**
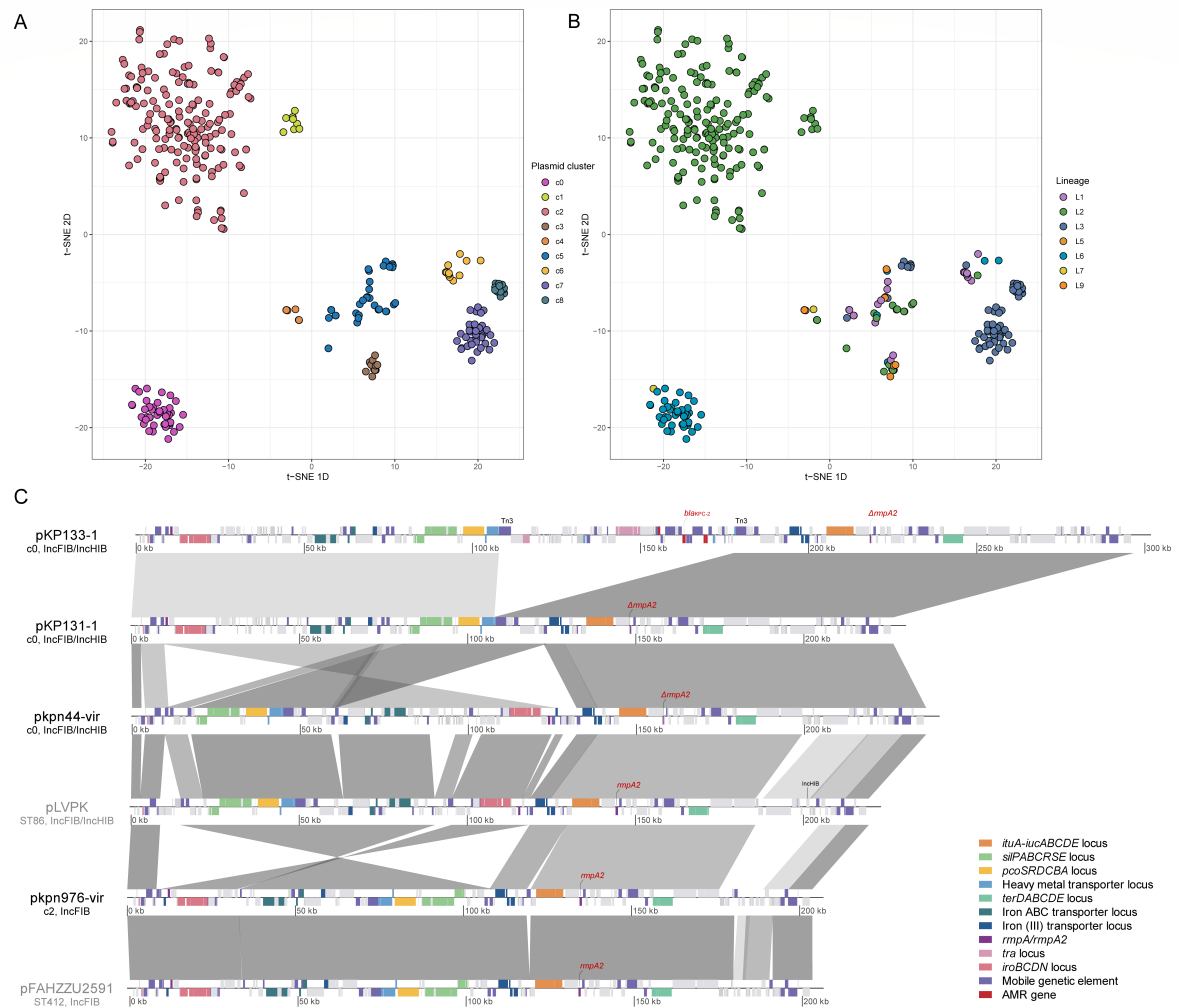
Clustering and structure of plasmid sequences. Virulence and carbapenemase-carrying plasmid sequences were clustered using the Mge-cluster tool, with colors assigned according to the plasmid cluster results (**A**) and the distinct ST268 lineages (**B**). (**C**) Linear sequence comparisons of representative plasmids illustrate the structural diversity among major virulence plasmid types.

Clustering analysis successfully categorized 400 of 428 plasmids, revealing distinct segregation between virulence and carbapenemase plasmids (Fig. 7A). Carbapenemase-carrying plasmids formed six major clusters (c3-c8), with cluster c5 (*n* = 50) being the largest, comprising mainly *bla*_NDM_ and *bla*_KPC_ carriers distributed across five lineages (L1-3, L6, and L9). Notably, cluster c7 (*n* = 40) contained exclusively *bla*_KPC-2_ and was restricted to lineage L3. Virulence plasmids clustered into three distinct groups (c0-c2): c2 was predominant (*n* = 199), followed by c0 (*n* = 47) and c1 (*n* = 12). Clusters c2 and c0 were exclusive to lineage L2, whereas cluster c1 was mainly concentrated in lineage L6 (Fig. 7B). Interestingly, two plasmids in cluster c6 carried only virulence determinants (*peg-344* and *rmpA*), suggesting potential acquisition of these genes by a carbapenemase plasmid backbone.

To further characterize these virulence plasmids, we performed complete genome sequencing of two representative strains from lineages L2 and L6. Analysis revealed that strain kpn976 (L2, cluster c1) harbored a 203-kbp IncFIB virulence plasmid (designated pkpn976-vir), while strain kpn44 (L6, cluster c0) carried a 236-kbp IncFIB/IncHI1B multi-replicon plasmid (designated pkpn44-vir). Comparative genomic analysis demonstrated that pkpn44-vir shared structural homology with the classic virulence plasmid pLVPK, albeit with a truncated *rmpA2* gene. In contrast, pkpn976-vir contained an intact *rmpA2* gene and exhibited closest similarity to pFAHZZU2591 plasmid (accession no. CP083752.1), with 98.37% similarity and 99.75% coverage. Compared to pLVPK, pkpn976-vir lacked the IncHI1B replicon and featured a genomic inversion within a ~100 kbp region (Fig. 7C).

Given the clinical significance of strains harboring both carbapenemase genes and hypervirulence determinants, we investigated their co-occurrence within the ST268 population. We identified 48 such strains, restricted to lineages L2 (85.4%, 41/48) and L6 (14.6%, 7/48). Notably, 95.8% (46/48) of these isolates maintained separate plasmids for resistance and virulence determinants, with only two instances of fusion plasmids carrying both traits. Both fusion plasmids originated from China, including the previously characterized pKP133-1 (accession no. CP097077) (66) belonging to plasmid cluster c0, which was formed through Tn3 transposition-mediated fusion of pKP131-1 (accession no. CP097080) and another *bla*_KPC-2_-carrying plasmid KP131-2 (accession no. CP097081; plasmid cluster c3).

### Lineage-specific genetic variations

To elucidate the evolutionary trajectories within the ST268 population, we identified lineage-specific nonsynonymous SNPs and gene acquisition events that distinguish isolates across different clusters. With the exception of the smaller lineages L8 and L10—which exhibited no lineage-specific nonsynonymous SNPs—the remaining lineages harbored a variable number of mutations, ranging from 2 in L9 to 49 in L4 (Table S4). Functional annotation revealed that these SNPs predominantly affected genes associated with diverse biological processes, including metabolic pathways, stress response, and virulence-related functions. For instance, lineage L1 displayed mutations in genes associated with carbohydrate metabolism and transport (*chbC* and *ascF*), iron acquisition (*efeB*), and cell wall modification (*amiC*). In lineage L2, SNPs were detected in genes related to cell wall synthesis (*ftsI* and *pbpC*), antibiotic resistance (*mdtM*, *eefB*, and *dinF*), iron acquisition (*fhuA*), and virulence regulation (*iscR* and *bcsB*). Meanwhile, lineage L3 exhibited mutations mainly in metabolic enzymes (*queE*) and DNA-binding proteins (*cbpA*), and lineage L6 harbored SNPs in genes involved in zinc transport (*zupT*), stress response (*msrA* and *pspB*), potassium transport (*kdpA*), and DNA repair (*dinB* and *exoX*).

Beyond SNPs, we also analyzed the accessory genome composition to further delineate lineage differentiation. t-SNE analysis of gene presence/absence patterns revealed distinct clustering aligned with the 10 defined lineages (L1–L10; Fig. S13A), and this pattern persisted after removal of plasmid-associated genes (data not shown), highlighting the accessory genome’s role in shaping population structure. Principal component analysis (PCA) corroborated this, with hypervirulent lineages L2 and L6 clustering tightly together, distinct from resistant lineages L1 and L3, which formed separate yet overlapping groups (Fig. S13B). These patterns suggest that accessory genome variation drives lineage-specific adaptations, potentially reflecting convergent responses to shared ecological pressures.

To explore the virulence-resistance dichotomy, we examined horizontally acquired genes in major lineages. Functional profiles showed no significant differences within resistant (L1 and L3) or hypervirulent (L2 and L6) groups. However, L1 and L3 were enriched in genes for inorganic ion transport and amino acid metabolism, while L2 and L6 favored trafficking and secretion genes (Fig. S14). These distinct acquisition profiles indicate convergent evolution, likely driven by niche-specific pressures favoring either enhanced resistance or virulence.

### Virulence of ST268 isolates in this study

**Fig 8:**
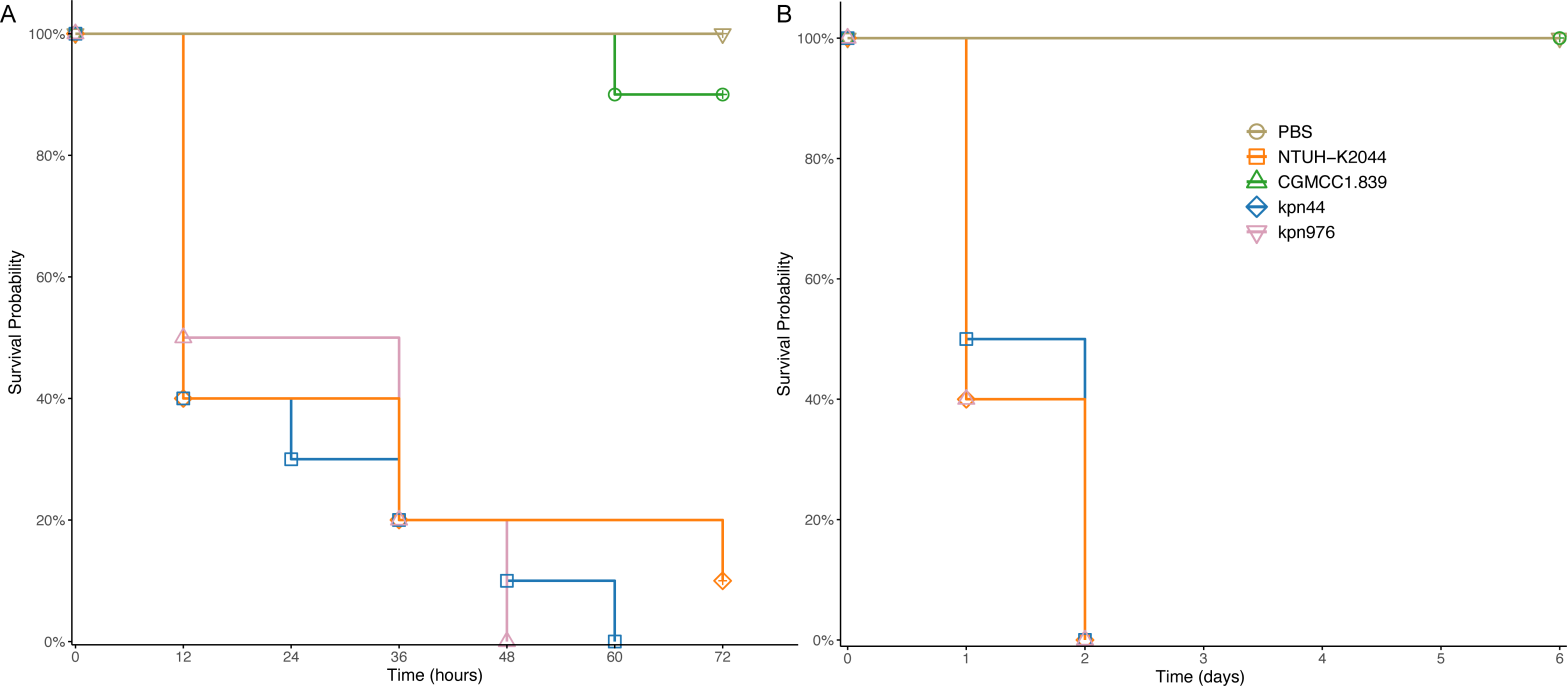
Virulence potential of ST268 isolates in the study. (**A**) Survival curves in the *G. mellonella* infection model. (**B**) Murine survival curves in the intraperitoneal infection model.

To assess the virulence potential of ST268 strains, we employed *G. mellonella* and mouse intraperitoneal infection models. In the *G. mellonella* assay (Fig. 8A), strains kpn976 (L2) and kpn44 (L6) exhibited mortality rates comparable to the highly virulent reference strain NTUH-K2044, while the environmental control strain CGMCC1.839 caused only one death after 60 hours. Similarly, in the mouse model (Fig. 8B), all mice infected with NTUH-K2044 or kpn976 or kpn44 (*n* = 10) succumbed within 2 days, whereas those infected with CGMCC1.839 survived up to 6 days when the experiment ended (*P* < 0.05). These virulence assays consistently demonstrated the high virulence of the ST268 strains kpn976 and kpn44, aligning with their carriage of hv markers.

## DISCUSSION

In this study, we performed a comprehensive population genomics analysis of 562 ST268 *K. pneumoniae* isolates collected from 44 countries, providing novel insights into the global diversity, epidemiology, and evolution of this sequence type. Phylogenetic analysis subdivided ST268 into 10 distinct lineages (L1–L10), with five major clades (*n* > 20) exhibiting contrasting virulence-resistance profiles: L2 and L6 were enriched in hypervirulence determinants, whereas L1 and L3 carried numerous carbapenemases. L9, despite its prevalence, displayed minimal virulence and resistance markers. Geographic clustering was evident, with L1 predominating in North America, L2 and L6 in Asia, and L3 in Europe—likely reflecting regional differences in antimicrobial use, infection control practices, and adaptation to local ecological niches. The identification of both localized outbreaks and sporadic ST268 infections across multiple countries highlights the need for enhanced global surveillance of this increasingly important sequence type.

Bayesian phylogeographic analysis places the likely origin of ST268 in Europe during the early 20th century. Notably, this appears inconsistent with the phylogeny, as the predominantly European lineage L3 displays less genetic diversity than the longer-branched Asian lineages L2 and L6 (Fig. 2A). This apparent paradox, however, dissolves upon a closer examination of the underlying population structure and lineage-specific histories. Lineage L9, an earlier-diverging and more diverse lineage of European origin, points to an ancient ST268 reservoir in Europe (Fig. 4; Fig. S8). By contrast, L3 likely represents a recent, hospital-adapted expansion whose apparent low diversity reflects over-sampling of outbreak strains rather than true evolutionary constraint. The extended branches of L2 and L6 may be better explained by accelerated substitution rates following their introduction into Asia—potentially driven by region-specific selective pressures—than by greater evolutionary age. This interpretation is consistent with their enrichment in virulence-associated functions and highlights how ST268 success is shaped by lineage-specific adaptation to distinct clinical and geographic contexts. Demographic reconstruction further suggests a long period of relative stability followed by a possible population expansion in the early 2000s, albeit with wide credible intervals. Although this expansion coincides with rising global antimicrobial consumption (67) and may have favored resistant lineages such as L1 and L3, antimicrobial pressure alone cannot account for the growth of largely susceptible hypervirulent lineages L2 and L6. Therefore, it is likely that a combination of factors—including selection for resistance in some ecological niches, selection for virulence in others, and increased global transmission opportunities—collectively underpins the contemporary success and clinical significance of ST268.

Our genomic analysis indicates limited recombination across the ST268 chromosome, which is largely clonal apart from several distinct recombination hotspots (Fig. 3B). This clonal stability is most evident at the K/O loci: the vast majority of isolates in all lineages retain the ancestral KL20 and O1/O2v1 types. The main exception is lineage L9, where local recombination events appear to have introduced additional K and O types. Thus, ST268 shows a generally stable K/O profile punctuated by occasional, lineage-specific replacement events—a pattern typical of many hvKp lineages and markedly different from the frequent antigen-switching reported for certain epidemic MDR clones (13). Notably, the ICE*Kp* element encoding yersiniabactin emerged as a significant hotspot for sequence variation, likely reflecting conjugative acquisition events rather than classical homologous recombination and substantially contributing to ST268’s genomic diversity. The strong lineage-specific associations of particular ICE*Kp* variants—ICE*Kp10* in L2, ICE*Kp3* in L3, and ICE*Kp4* in L1—suggest that these mobile elements play a decisive role in shaping ST268’s population structure. The heterogeneous distribution of ICE*Kp* types across isolates indicates multiple independent acquisition and loss events throughout ST268’s evolutionary history. Furthermore, the distribution of lineage-specific nonsynonymous SNPs revealed distinct functional patterns: L1 accumulated variants affecting carbohydrate metabolism, L2 showed changes in cell wall synthesis and antibiotic resistance genes, while L6 harbored mutations in stress response and DNA repair pathways. These divergent mutational profiles suggest lineage-specific genetic differentiation—and potentially functional divergence—that may be shaped by varying ecological or clinical contexts.

A striking finding of our study is the highly lineage-specific distribution of resistance and virulence determinants across ST268. Lineages L2 and L6 harbored significantly more virulence genes, including hypervirulence markers, while L1 and L3 predominantly carried carbapenemase genes, and other lineages showed a stronger association with CTX-M ESBLs (Fig. 5D). The scarcity of resistance-conferring mutations across all lineages suggests that horizontal gene transfer, rather than mutational evolution, drives resistance acquisition in ST268. This distribution pattern indicates a potential evolutionary trade-off between resistance and virulence capabilities. Supporting this hypothesis, our gene acquisition analysis revealed that L2 and L6 preferentially acquired genes related to intracellular trafficking and secretion systems—functions critical for virulence expression—while L1 and L3 favored genes involved in inorganic ion transport, which likely facilitate cellular homeostasis in antibiotic-rich environments. From a public health perspective, this dichotomy creates region-specific clinical challenges, as geographic distribution of lineages corresponds to distinct virulence-resistance profiles, necessitating tailored surveillance and control strategies.

Plasmid analysis revealed remarkable genomic plasticity within ST268, particularly evident in the diverse repertoire of carbapenemase-encoding plasmids. The *bla*_KPC_ gene was associated with 13 distinct replicon types, a diversity greater than that of other carbapenemases in this data set. This highlights its significant contribution to the dissemination of carbapenem resistance within the ST268 lineage. Such plasmid promiscuity is consistent with the known association of *bla*_KPC_ with mobile genetic elements like the Tn*4401* transposon, which facilitate its transfer across diverse plasmid backbones (68). This extensive plasmid heterogeneity poses significant challenges for infection control, as varying plasmid backgrounds may confer distinct fitness advantages, stability profiles, and expression patterns, potentially contributing to treatment failures and outbreak persistence. Beyond resistance, virulence plasmids in lineages L2 and L6 showed lineage-specific clustering (c0 and c1) and replicon types (IncFIB and IncFIB/IncHI1B), aligning with their phylogenetic separation. Notably, a substantial proportion of L2 isolates co-harbored carbapenemase genes and hypervirulence determinants, albeit typically on separate plasmids, resulting in clinically challenging strains combining high resistance and enhanced virulence. More concerning was the identification of rare fusion plasmids in two isolates (L2 and L6) from China, carrying both carbapenem resistance and virulence determinants on a single mobile element. While infrequent in ST268, such fusion events represent a worrisome evolutionary development, as they enable simultaneous transfer of both trait complexes through a single horizontal gene transfer event, potentially accelerating the emergence of hypervirulent-resistant strains.

Our study has limitations. Primarily, our reliance on database-derived samples introduces inevitable selection biases, as isolates with pronounced resistance or virulence are more likely to be sequenced. This may lead to an overestimation of the overall resistance and virulence levels attributed to the ST268 clone, whose true phenotypic spectrum within the broader population is likely more diverse. This reliance also skews the data set geographically, with Asia, North America, and Europe overrepresented compared to under-sampled regions like Africa and South America. Such an imbalance not only obscures a full picture of ST268’s global distribution but may also generate a biased understanding of its evolutionary pathways, potentially missing unique lineages or adaptive mechanisms that evolved independently in under-sampled regions. Furthermore, of the 562 isolates analyzed, only four were self-collected, with the rest sourced from public databases; notably, these self-collected strains belong exclusively to the highly virulent lineages L2 and L6. This focus limits experimental insights into other lineages, meaning our conclusions regarding their functional traits—such as the presumed lower virulence of non-L2/L6 lineages—remain genomic inferences that lack robust validation from *in vitro* and *in vivo* assays. Additionally, our analysis of lineage-specific nonsynonymous SNPs is purely descriptive; we did not perform formal selection tests to determine whether these variants are under positive selection. This limitation prevents us from conclusively linking the observed mutations to adaptive evolution. Finally, plasmid reconstruction via MOB-suite, while advanced (69), is not immune to the inherent shortcomings of bioinformatics tools, especially when dealing with fragmented short-read assemblies. This could lead to an underestimation of plasmid diversity or the misassignment of carbapenemase and virulence gene linkages. In particular, a key finding of our study—the prevalence of fusion plasmids carrying both resistance and virulence genes—is likely underestimated. Therefore, this figure should be interpreted as a conservative estimate, and the conclusions regarding these genetic linkages should be treated with caution. To address these gaps, future research should prioritize broader geographic sampling, incorporate systematic functional experiments, and leverage long-read sequencing to resolve complex plasmid architectures, thereby strengthening and extending our observations.

In conclusion, this comprehensive genomic investigation delineates distinct evolutionary pathways and adaptation strategies within *K. pneumoniae* ST268. Our results emphasize clear lineage-specific differentiation in antimicrobial resistance and virulence profiles, driven by recombination hotspots, plasmid dynamics, and adaptive gene acquisitions. The rapid global spread of ST268 since the early 2000s underscores the urgency of implementing precise surveillance and containment measures against this high-risk pathogen.

## Data Availability

The genome sequences of the ST268 isolates from our study are available in the GenBank database under BioProject PRJNA1243865.

## SUPPLEMENTAL MATERIAL

Supplemental figures aac.00703-25-s0001.pdf Fig. S1 to S14.

Supplemental tables aac.00703-25-s0002.xlsx Tables S1 to S6.

ASM does not own the copyrights to Supplemental Material that may be linked to, or accessed through, an article. The authors have granted ASM a non-exclusive, world-wide license to publish the Supplemental Material files. Please contact the corresponding author directly for reuse.
