# Supplementary material for: Lineage-specific evolution and resistance-virulence divergence in Klebsiella pneumoniae ST268: a global population genomic analysis
Source: Antimicrob Agents Chemother. 2025 Sep 15;69(10):e00703-25. doi: 10.1128/aac.00703-25 (PMC12486848; doi:10.1128/aac.00703-25)
Supplement: Supplemental figures — Fig. S1 to S14. [file aac.00703-25-s0001.pdf]

Supplementary Information

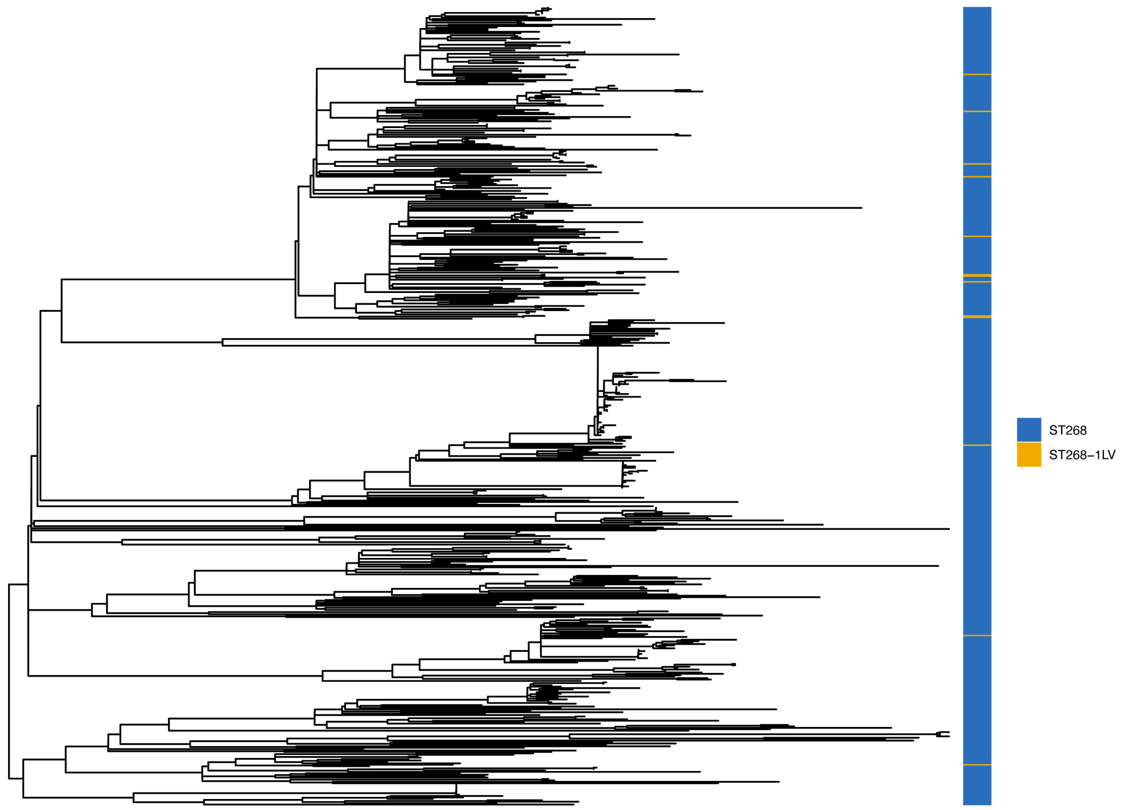

Figure S1. Maximum likelihood phylogeny for the 562 isolates collected in this study. ST268 and one-locus variants (ST268_1LV) isolates are shown in different colors. ST268_1LV isolates are dispersed throughout the tree.

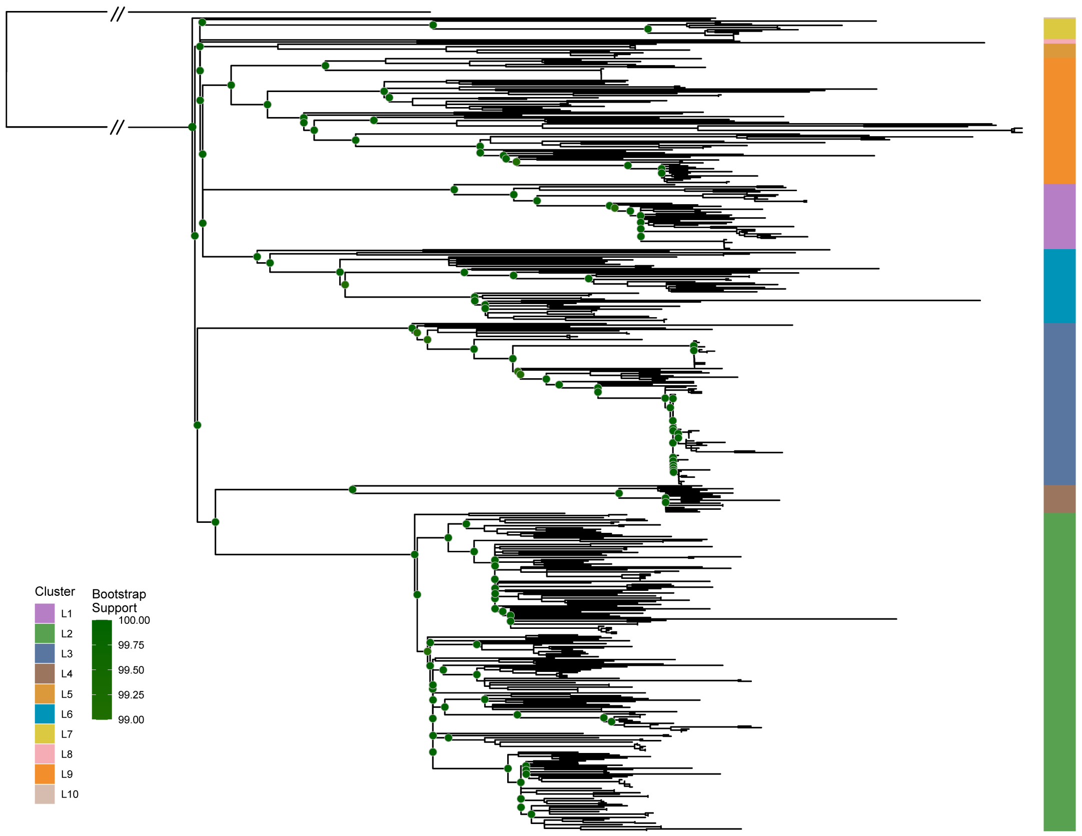

Figure S2. Maximum-likelihood phylogenetic tree of 562 *Klebsiella pneumoniae* ST268 genomes, rooted using NTUH-K2044 (ST23) as outgroup. Double slash (//) indicates artificial shortening of the outgroup branch for visualization. Bootstrap support values are shown for internal nodes with >10 descendant tips. The heatmap displays BAPS level 1 clustering results, with colors representing the ten phylogenetic lineages.

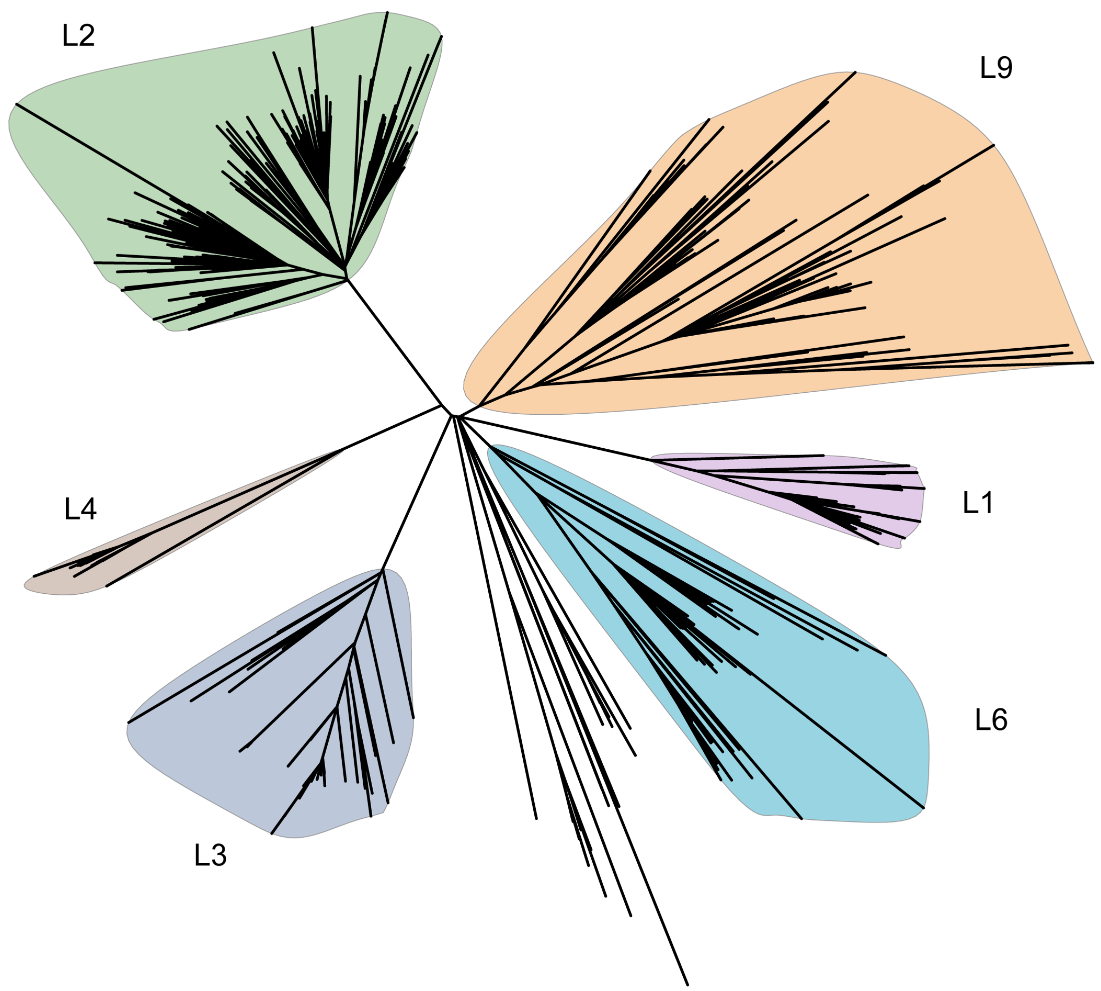

Figure S3. Unrooted tree shows the evolutionary distance between lineages. six clusters that can be clearly identified based on the topology of the phylogenetic tree are highlighted.

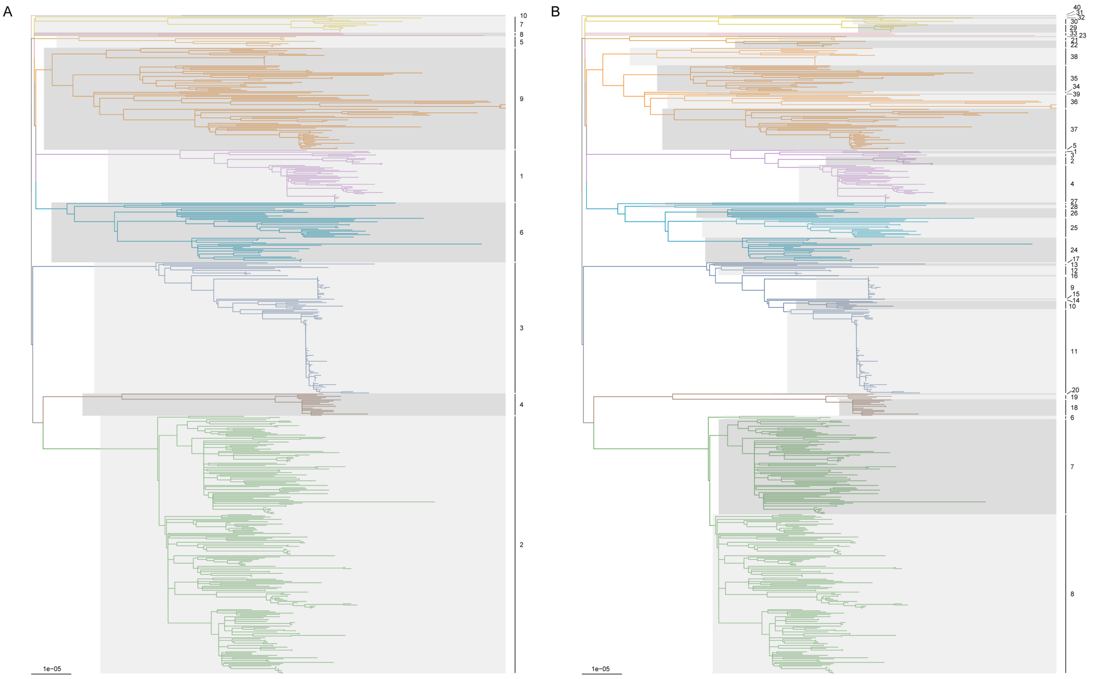

Figure S4. FastBaps clustering of ST268 genomes at levels 1 and 2. The phylogenetic tree was generated as in Fig. 2B. Branches are colored by level 1 clusters, and clades shaded by: (A) level 1, (B) level 2. Numbers on the right indicate the corresponding FastBaps cluster assignments.

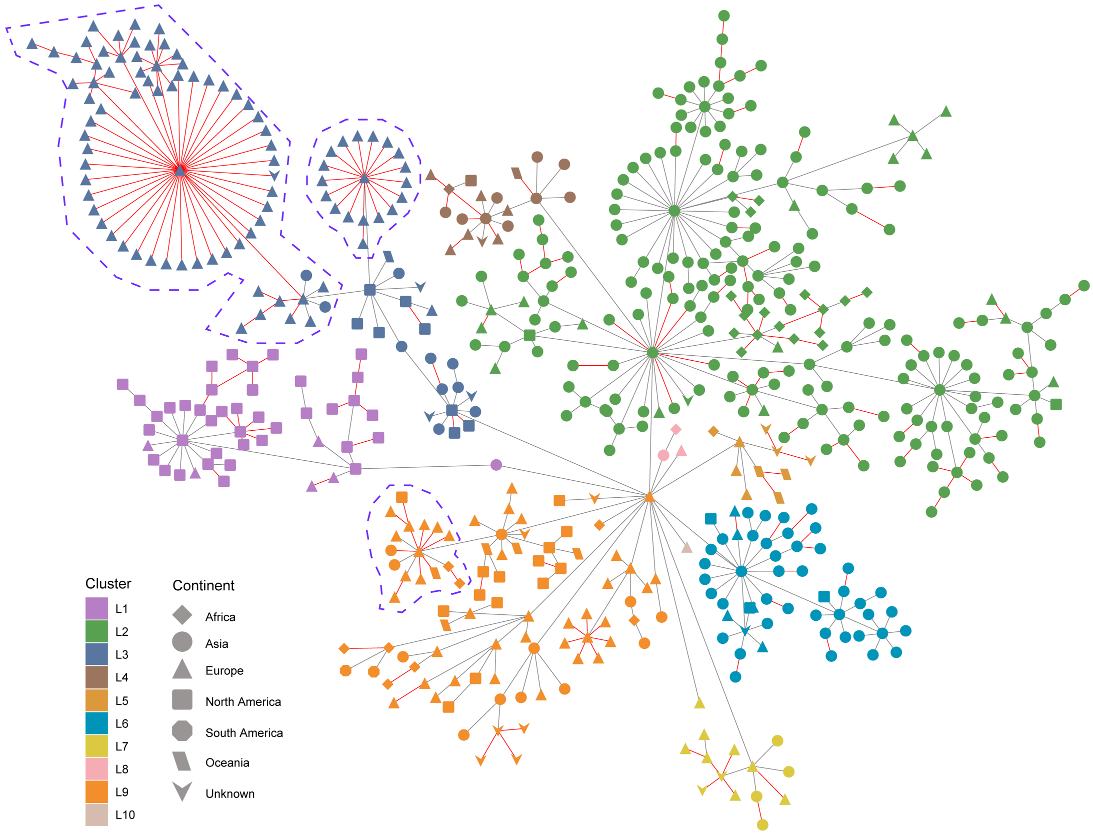

Figure S5. Minimum spanning tree (MST) based on pairwise SNP distances. Each node represents a single isolate. Node color indicates lineage, and red lines connect isolates with ≤16 SNPs, indicating potential transmission events. Node shapes represent the continent of origin. The three major transmission clusters described in the text are enclosed by irregular dashed lines.

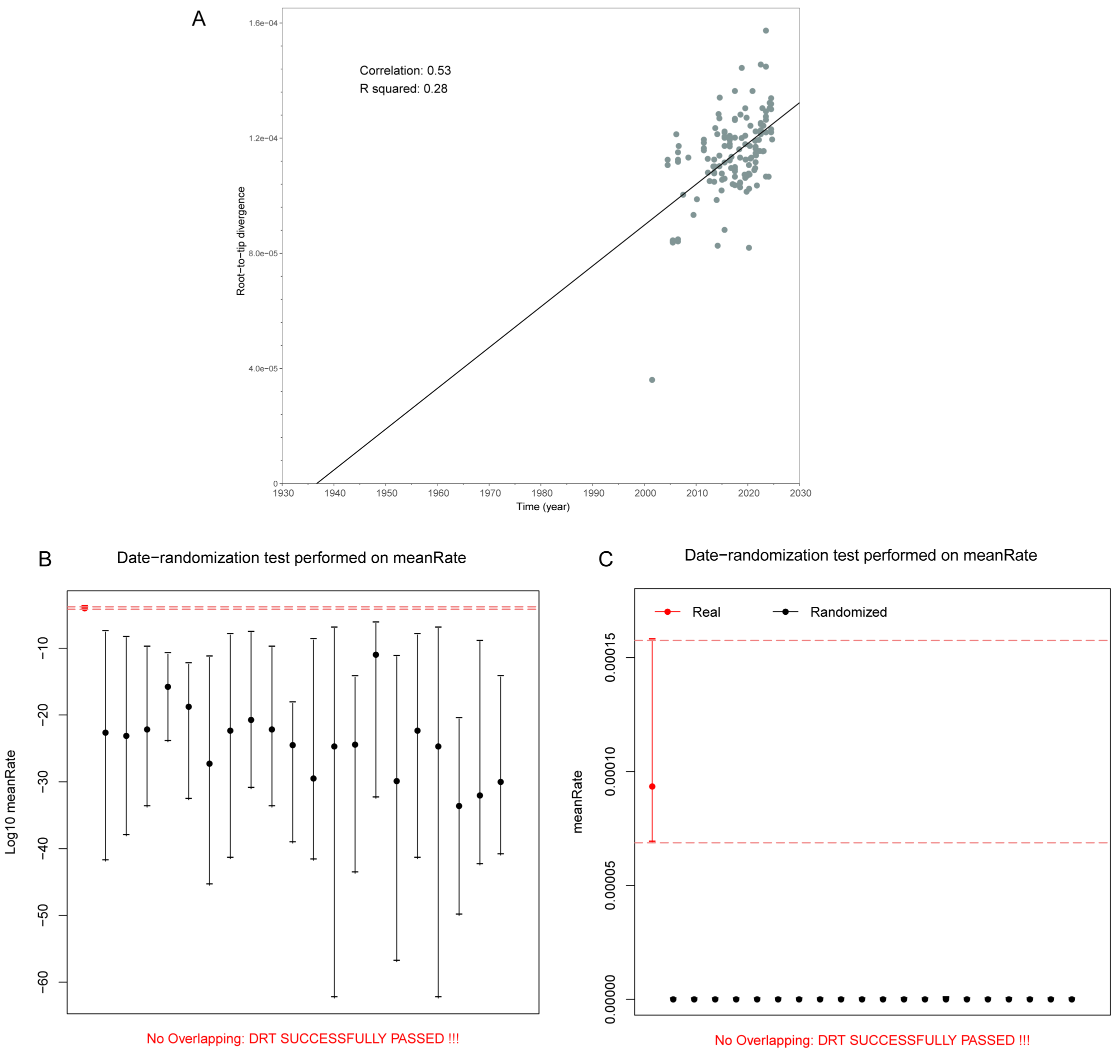

Figure S6. Root-to-tip analysis (A) and date-randomization test (B). In the date- randomization tests, the 95% confidence intervals (CI) for the estimates of the clock rate (left graph) and root height (right graph) of the observed data (in red) do not overlap with the confidence intervals for the estimates obtained from the randomized sets (in black, 20 independent runs), indicating strong temporal signals. Data are presented as mean and 95% CI.

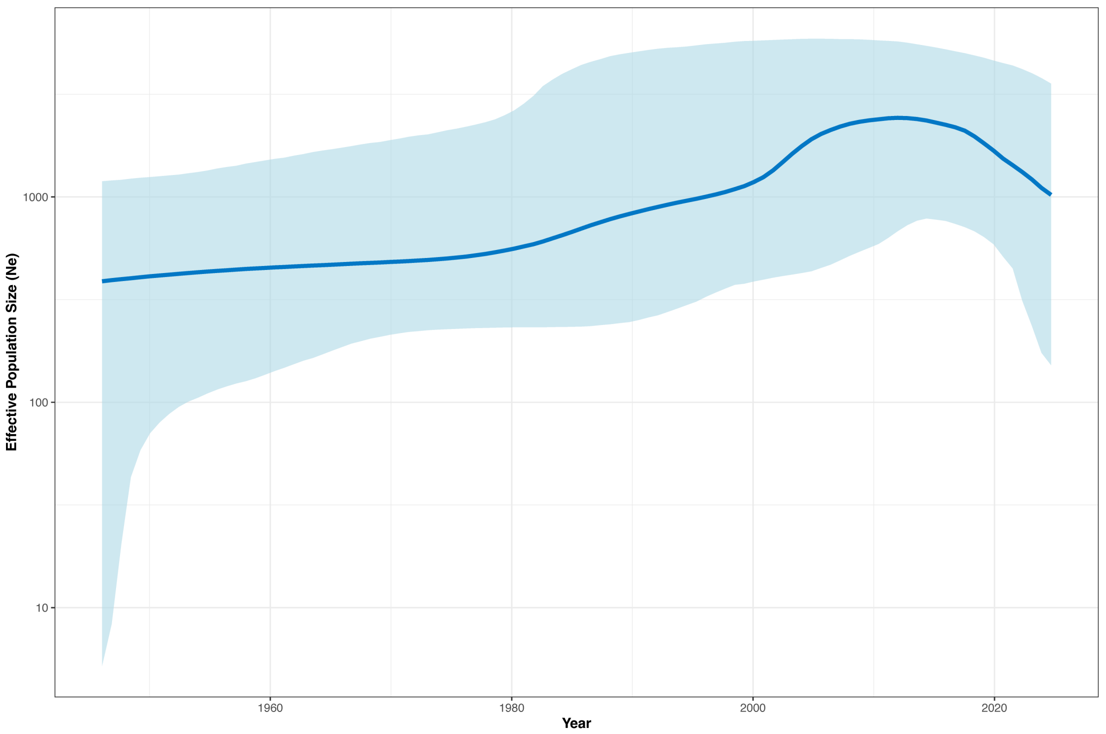

Figure S7. Bayesian skyline plots showing the historical changes in the effective population size of ST268 isolates. Solid lines represent the medians of estimated effective population sizes. Dashed lines and shadings indicate the upper and lower bounds of the 95% highest posterior density intervals.

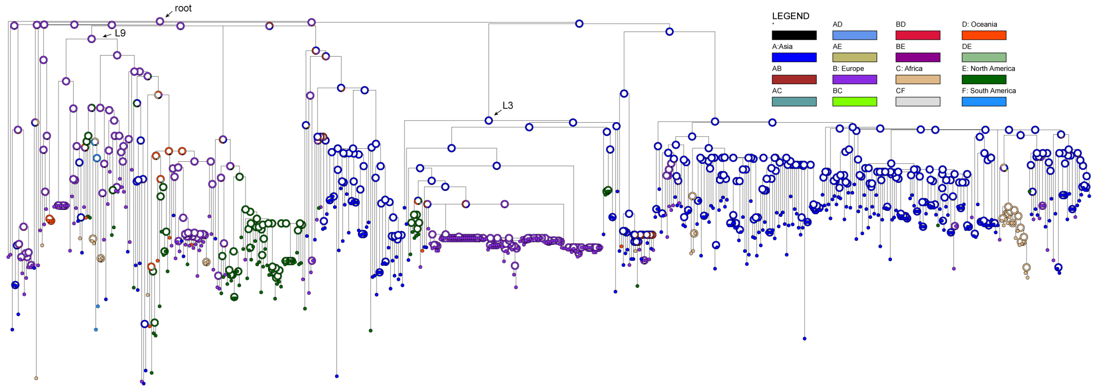

Figure S8. Phylogeographic analysis of the ST268 isolates based on the Bayestraits MCMC model.

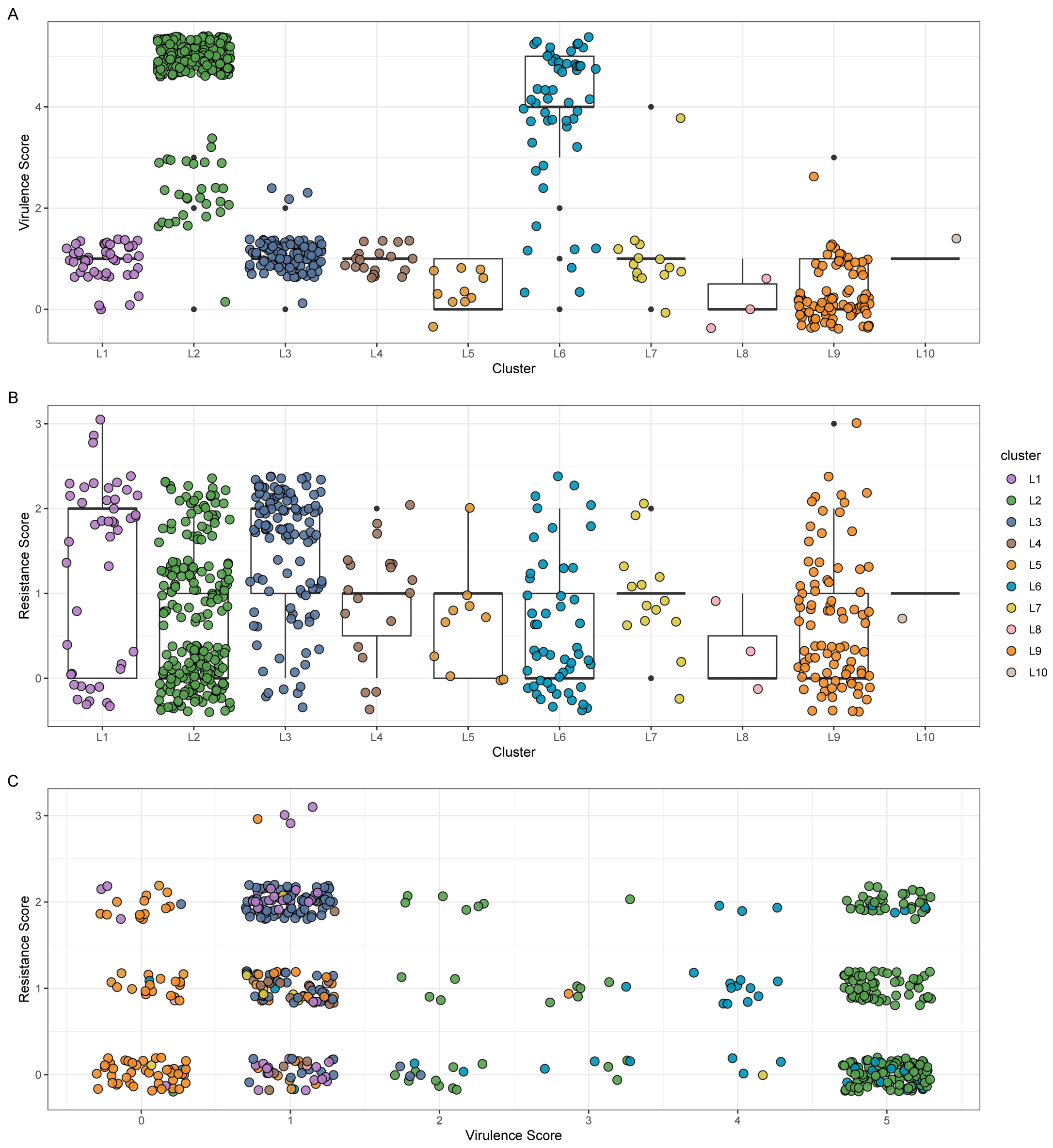

Figure S9. Distribution of ST268 lineages based on kleborate virulence and resistance scores with each point representing a strain. (A) Virulence scores. (B) Resistance scores. (C) Combined virulence and resistance scores. Points are colored by lineage.

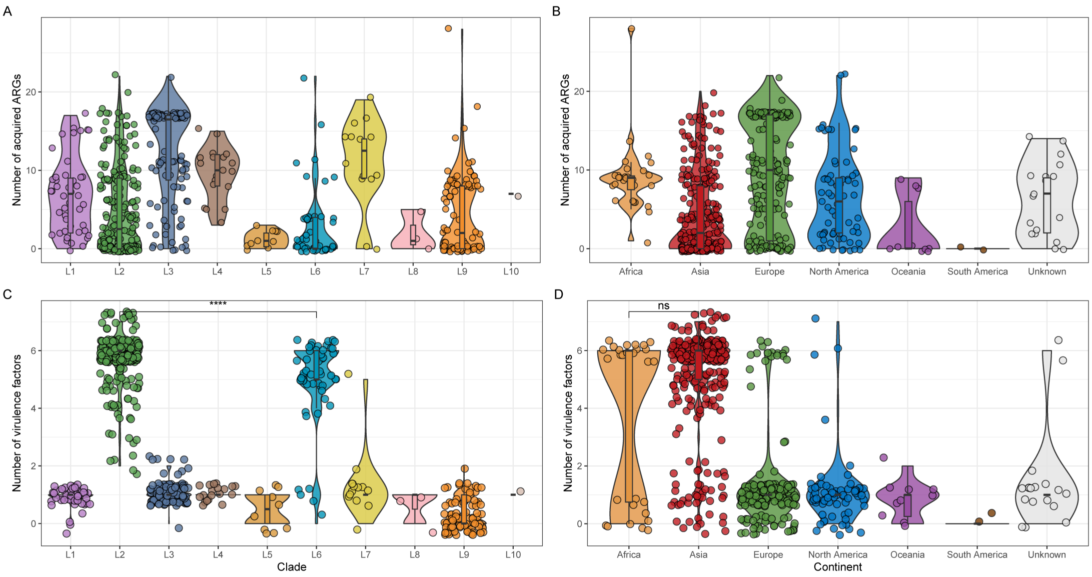

Figure S10. AMR and virulence genes in ST268 isolates. Number of acquired AMR genes per isolate in different lineages (A) and continents (B). Number of non-core virulence factors per isolate in different lineages (C) and continents (D).

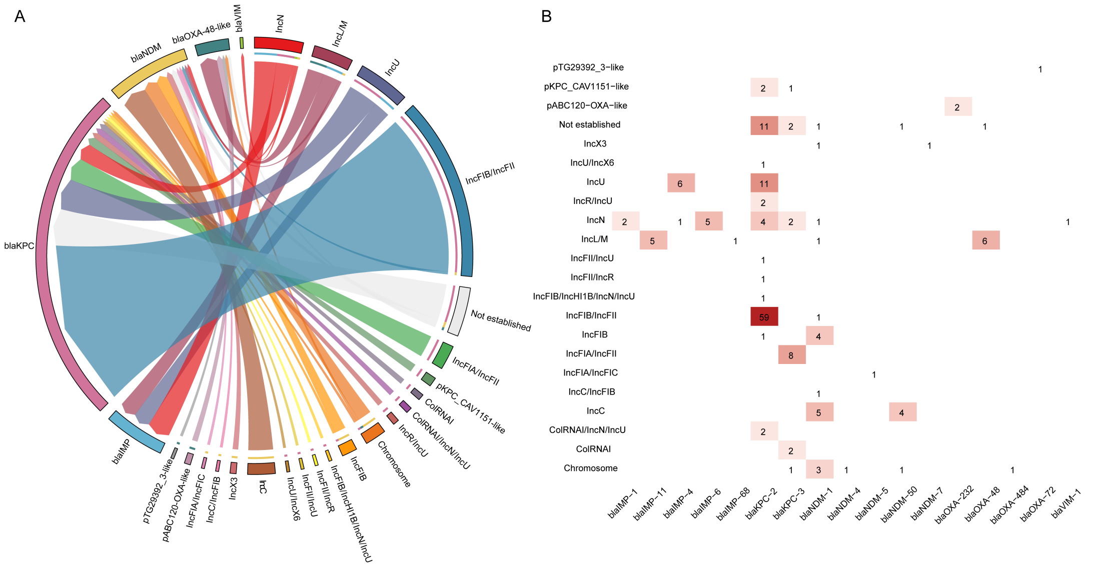

Figure S11. Relationship between carbapenem-resistant plasmids and replicon types. (A) Distribution of replicon types associated with different gene categories. (B) Replicon types corresponding to each specific resistance gene.

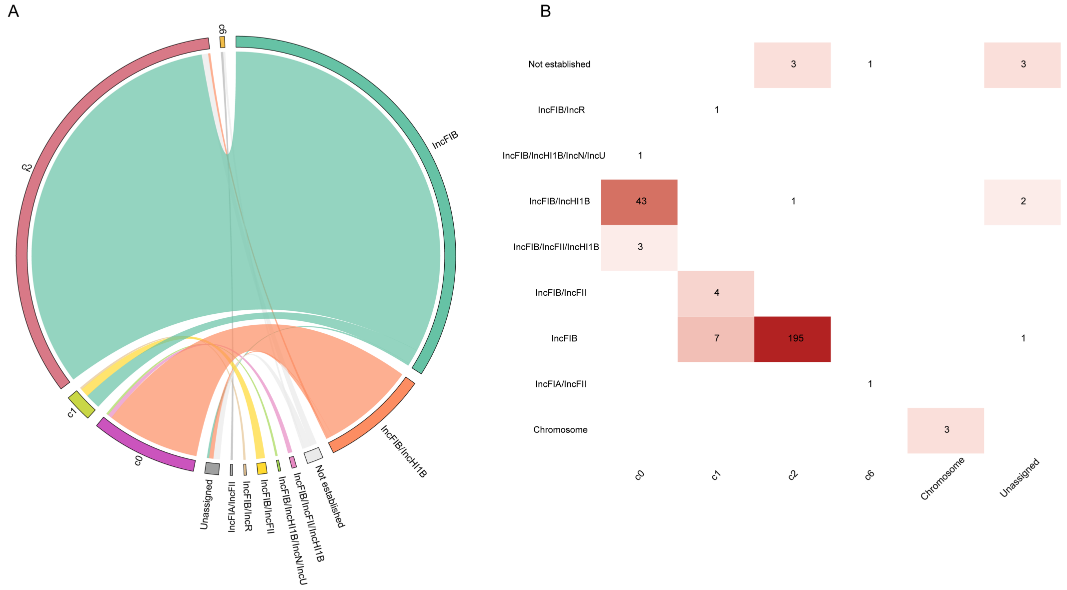

Figure S12. Relationship between virulence plasmid clusters and replicon types. The association is visualized using a chord diagram (A) and a heatmap (B).

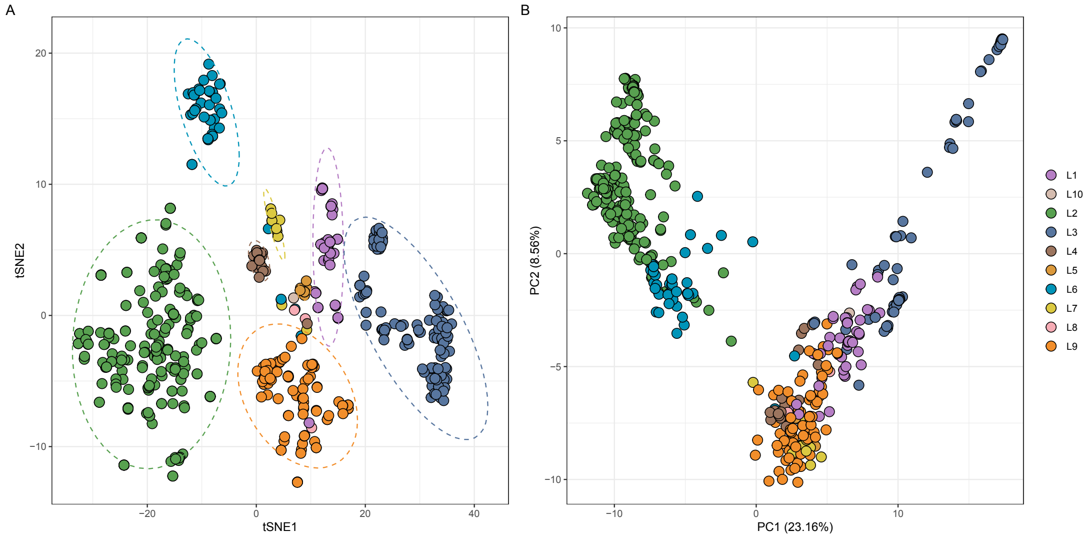

Figure S13. tSNE (A) and PCA (B) based on the accessory gene content matrix of 562 isolates, where each circle represents an isolate, colored according to its clade.

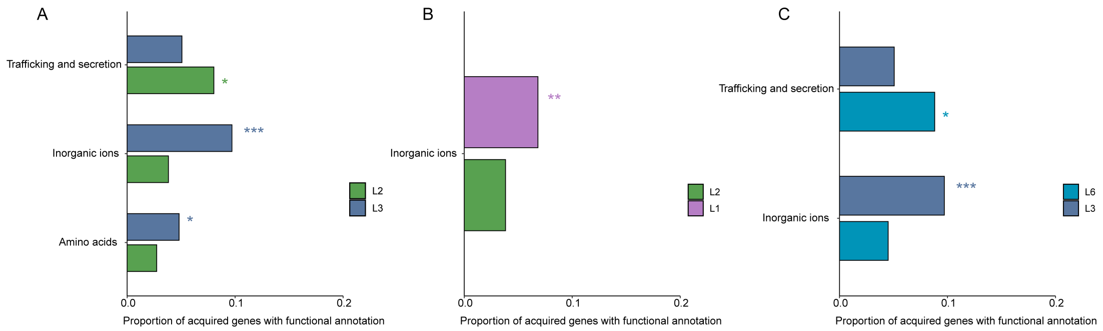

Figure S14. Gene gain events across ST268 lineages L1, L2, L3, and L6. Proportion of acquired genes with functional annotations based on the COG database, inferred through maximum parsimony ancestral genome reconstruction. Only COG categories showing significant differences in gene acquisition among these lineages are displayed. *$p < 0.05$; **$p < 0.01$; ***$p < 0.001$.
